# Pooled analysis of 3,741 stool metagenomes from 18 cohorts for cross-stage and strain-level reproducible microbial biomarkers of colorectal cancer

**DOI:** 10.1038/s41591-025-03693-9

**Published:** 2025-06-03

**Authors:** Gianmarco Piccinno, Kelsey N. Thompson, Paolo Manghi, Andrew R. Ghazi, Andrew Maltez Thomas, Aitor Blanco-Míguez, Francesco Asnicar, Katarina Mladenovic, Federica Pinto, Federica Armanini, Michal Punčochář, Elisa Piperni, Vitor Heidrich, Gloria Fackelmann, Giulio Ferrero, Sonia Tarallo, Long H. Nguyen, Yan Yan, Nazim A. Keles, Bilge G. Tuna, Veronika Vymetalkova, Mario Trompetto, Vaclav Liska, Tomas Hucl, Pavel Vodicka, Beatrix Bencsiková, Martina Čarnogurská, Vlad Popovici, Federica Marmorino, Chiara Cremolini, Barbara Pardini, Francesca Cordero, Mingyang Song, Andrew T. Chan, Lisa Derosa, Laurence Zitvogel, Curtis Huttenhower, Alessio Naccarati, Eva Budinska, Nicola Segata

**Affiliations:** 1 Department CIBIO, University of Trento, Trento, Italy; 2 Department of Biostatistics, Harvard T.H. Chan School of Public Health, Boston, MA USA; 3 Harvard Chan Microbiome in Public Health Center, Harvard T.H. Chan School of Public Health, Boston, MA USA; 4 Infectious Disease and Microbiome Program, Broad Institute of MIT and Harvard, Cambridge, MA USA; 5 IEO, European Institute of Oncology, IRCCS, Milan, Italy; 6 Department of Clinical and Biological Sciences, University of Torino, Torino, Italy; 7 Department of Computer Science, University of Torino, Torino, Italy; 8 Italian Institute for Genomic Medicine (IIGM), c/o IRCCS Candiolo, Candiolo, Italy; 9 Candiolo Cancer Institute, FPO-IRCCS, Candiolo, Italy; 10 Clinical and Translational Epidemiology Unit, Massachusetts General Hospital, Boston, MA USA; 11 Department of Medical Biology, School of Medicine, Yeditepe University, Istanbul, Turkey; 12 Graduate School of Natural and Applied Sciences, Yeditepe University, Istanbul, Turkey; 13 Department of Biophysics, School of Medicine, Yeditepe University, Istanbul, Turkey; 14 Department of Molecular Biology of Cancer, Institute of Experimental Medicine of the Czech Academy of Sciences, Prague, Czech Republic; 15 Institute of Biology and Medical Genetics, 1st Medical Faculty, Charles University, Prague, Czech Republic; 16 Biomedical Center, Faculty of Medicine in Pilsen, Charles University, Prague, Czech Republic; 17 Department of Colorectal Surgery, Clinica S. Rita, Vercelli, Italy; 18 Department of Surgery, University Hospital and Faculty of Medicine in Pilsen, Charles University, Prague, Czech Republic; 19 Department of Hepatogastroenterology, Institute for Clinical and Experimental Medicine, Prague, Czech Republic; 20 Masaryk Memorial Cancer Institute, Brno, Czech Republic; 21 RECETOX, Faculty of Science, Masaryk University, Brno, Czech Republic; 22 Department of Translational Research and New Technologies in Medicine and Surgery, University of Pisa, Pisa, Italy; 23 Departments of Epidemiology and Nutrition, Harvard T.H. Chan School of Public Health, Boston, MA USA; 24 Gustave Roussy, Villejuif, France; 25 Faculté de Médecine, Université Paris-Saclay, Kremlin-Bicêtre, France; 26 Center of Clinical Investigations for In Situ Biotherapies of Cancer (BIOTHERIS), INSERM CIC1428, Villejuif, France; 27 Institut National de la Santé Et de la Recherche Médicale (INSERM) U1015, Equipe Labellisée—Ligue Nationale contre le Cancer, Villejuif, France

**Keywords:** Metagenomics, Microbiome, Diagnostic markers

## Abstract

Associations between the gut microbiome and colorectal cancer (CRC) have been uncovered, but larger and more diverse studies are needed to assess their potential clinical use. We expanded upon 12 metagenomic datasets of patients with CRC (*n* = 930), adenomas (*n* = 210) and healthy control individuals (*n* = 976; total *n* = 2,116) with 6 new cohorts (*n* = 1,625) providing granular information on cancer stage and the anatomic location of tumors. We improved CRC prediction accuracy based solely on gut metagenomics (average area under the curve = 0.85) and highlighted the contribution of 19 newly profiled species and distinct *Fusobacterium nucleatum* clades. Specific gut species distinguish left-sided versus right-sided CRC (area under the curve = 0.66) with an enrichment of oral-typical microbes. We identified strain-specific CRC signatures with the commensal *Ruminococcus bicirculans* and *Faecalibacterium prausnitzii* showing subclades associated with late-stage CRC. Our analysis confirms that the microbiome can be a clinical target for CRC screening and characterizes it as a biomarker for CRC progression.

## Abstract (web summary)

An analysis of 18 metagenomic datasets of individuals with colorectal cancer, adenomas and healthy controls yields improved cancer prediction accuracy based solely on gut metagenomics, as well as the identification of new species associated with the development of cancer.

## Main

CRC is the third most frequent and the second most lethal tumor type worldwide^1^. It has a 30% higher incidence in men^2^ and 60–65% of all CRC cases occur in individuals with no previous family history (sporadic cancers)^3^. Only 40% of cases are diagnosed before metastasis^2^, with highest survival rates when the tumor is diagnosed at an early stage and a 5-year survival rate for stage IV for colon and rectal cancer of 11% and 15%, respectively^4^. CRC originates in the epithelial layer of either the proximal or distal colon plus rectum^5^, usually referred to as right- and left-sided CRC, respectively. Progression from benign precursor lesion (adenoma) to a malignant tumor (carcinoma), termed the adenoma–carcinoma sequence, may take several years^6^ and is characterized by an accumulation of mutations in tumor cells^5^, impairment in the gut mucosal barrier and intestinal inflammation^7,8^.

Interest in the tumor microenvironment has increased alongside advances in distinguishing tumor histological features and expression patterns of CRC^9^, with the gut microbiome suggested as another important hallmark of cancer^9^. Specific microbes have been proposed as major contributors to carcinogenesis, particularly *pks*^+^ *Escherichia coli* and *Fusobacterium nucleatum*^10,11^. Several individual cohort studies and earlier meta-analyses have observed distinct microbiome signatures in patients with CRC when compared with patients with adenomas or healthy controls^12–17^, consistently across different countries and cohorts^18–20^. A few noteworthy metagenomic studies also interrogated microbiome changes along the adenoma–carcinoma sequence and according to primary neoplasia location^15,21^, and links between CRC and oral species have been suggested^15^. Further evidence points toward the enrichment of oral-typical microbes (at the genus level^21^) and of oral biofilm-forming species^22^ in the gut metagenomes of patients with proximal CRC. However, no metagenomic studies have gone beyond characterizing already well-known strain-specific factors influencing CRC risk (for example, *pks* island, fragilysin), and no untargeted searches for subspecies and strain-level genomic associations with CRC phenotypes are available. These gaps in the state-of-the-art currently limit the microbiome’s potential to be used as a screening tool in clinical settings.

Here, we investigated gut microbiome composition along the adenoma–carcinoma sequence and across different primary tumor locations using a meta-analytical approach comprised of an unprecedented number of cohorts (12 public studies and 6 new cohorts generated in this study) and samples (2,116 from public studies and 1,625 from our new CRC cohorts). We also used new computational, statistical and machine learning (ML) strategies to achieve higher profiling resolution extended to previously unknown species and differentiated clades of *F. nucleatum*^23^.

## Results

### An expanded metagenomic study population for CRC

**Fig. 1:**
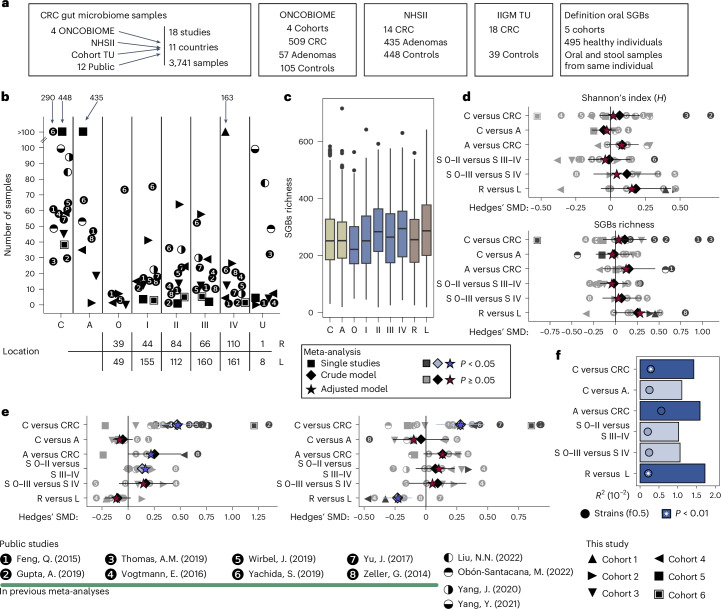
Overall and oral taxa-specific gut microbial diversity were significantly different according to CRC status, stage and primary tumor location. **a**, Overview of the cohorts (*n* = 18) and sample sizes (*n* = 3,741) according to case-control, cancer stage and primary tumor location, along with the cohorts used to define oral-typical species. **b**, Number of samples available from each CRC stage and the two primary tumor locations. Symbols indicate the cohort. **c**, Sample microbiome richness at each stage and primary tumor location. Box plots represent the within-category microbial richness distribution summarized by the first and third quartiles as hinges of the box, the median and whiskers extending to the largest or smallest value not exceeding 1.5× the interquartile range from the two ends of the box, with data beyond these values plotted individually as outliers. **d**, Meta-analyzed SMDs of the associations between alpha-diversity (Shannon diversity (upper) and SGB richness (lower)) and all paired comparisons. The 95% CIs for each meta-analysis model are indicated by a horizontal line. *P* values were computed via two-tailed *t*-test. Significant associations (*P* < 0.05) are indicated by a light blue diamond. SMD values corrected for age, sex and BMI (Methods) are indicated by a star (blue when *P* < 0.05). No correction for multiple hypothesis testing was performed. **e**, Meta-analysis of the association between the cumulative relative abundance of oral species (oral-to-gut score) and all paired comparisons (left) and between the number of oral species (oral-to-gut richness) and all paired comparisons (right). Symbols and axes are similar to **d**. *P* values were computed via two-tailed *t*-test. No correction for multiple hypothesis testing was performed. SMD values corrected for age, sex and BMI are indicated by a star. **f**, PERMANOVA (stratified by dataset) derived *R*^2^ according to CRC stage and primary tumor location, computed via adonis2 (Methods) on Bray–Curtis distances. Comparisons with *P* < 0.01 are highlighted in dark blue. Circles indicate the *R*^2^ explained by strain-level microbial features, and comparisons with *P* < 0.01 are marked with an asterisk. The text f0.5 denotes the feature set defined in the Methods section ‘Strain-level feature identification’. A, adenoma; C, control; L, left-sided; R, right-sided; 0, stage 0; I, stage I; II, stage II; III, stage III; IV, stage IV; U, stage not available.

We established a large and diverse set of gut metagenomic cohorts associated with sporadic CRC and with information on CRC stage (stages 0–IV) and primary tumor location (right-sided or left-sided). To this end, we sequenced 1,625 new stool metagenomes from 6 previously unpublished CRC cohorts (Methods) and integrated them with 2,116 stool metagenomes from 12 public studies. In total, we leveraged 1,471 samples from patients with CRC (1,191 with staging information and 989 with primary tumor location information), 702 from patients with colorectal adenoma and 1,568 from control participants, from 16 case-control and two CRC-only studies (Supplementary Tables 1 and 2). Four of the six newly sequenced cohorts (cohorts 1–4, *n* = 671) are part of the European ONCOBIOME initiative (Methods and ‘Data Availability’), whereas the fifth (cohort 5) is part of the Micro-N Nurses’ Health Study II (NHSII) (*n* = 897)^24^. Cohort 6 included stool samples from CRC cases and controls (*n* = 18 and 39, respectively) from the Umraniye Training and Research Hospital and the Department of Medical Biology, Yeditepe University (Istanbul, Turkey). Considering the 3,741 metagenomes in the 18 integrated datasets, we gathered 94 stool metagenomes from patients with stage 0 CRC or carcinoma in situ, and more than 250 for each single stage from stage I to stage IV. In total, 344 samples were from individuals whose primary tumors originated in the right colon (cecum, ascending and transverse colon (10 cohorts)) and 645 samples were from patients whose primary tumors originated in the left colon and rectum (11 cohorts) (Fig. 1a,b and Supplementary Tables 1 and 2a). In addition, cohort 1 includes patients with stage IV CRC with either resected primary tumor (*n* = 68) or in situ primary tumor (*n* = 95).

Samples were profiled using MetaPhlAn 4 (ref. ^25^), which leverages species-level genome bins (SGB)^26^ to enumerate and quantify characterized (known SGBs (kSGBs) having at least one cultivated reference) and uncharacterized species (unknown SGBs (uSGbs) lacking cultured representatives). In total, we detected 3,866 bacterial, 15 eukaryotic and 23 archaeal SGBs. Some bacterial species spanned multiple SGBs, as was the case for CRC-associated *F. nucleatum* species for which five SGBs described known and unknown subspecies found by MetaPhlAn 4 (that is, SGB6001, SGB6007, SGB6011, SGB6013, SGB6014), with SGB6007 and SGB6013 recently independently investigated^23^ and corresponding to *F. nucleatum* subspecies *animalis (Fna*) clade 2 (C2) and *Fna* clade 1 (C1)^23^. To test the relevance of the presence and overall abundance of oral microbial species in the CRC gut ecosystem, we defined a panel of typically oral SGBs. These were defined on an independent set of 990 matched oral and stool samples from 495 healthy individuals in 5 public microbiome studies^27^ (Methods). In particular, we considered oral SGBs to be those prevalent (>20%) in the oral microbiome but not (<5%) in the gut microbiome (Methods and Supplementary Table 3).

Functional profiles were also generated with HUMAnN 3.6 (ref. ^28^), and used for a comprehensive analysis on UniRef90 (UR90) gene profiles and corresponding functional grouping according to MetaCyc Pathways, Enzyme Commission (EC) and Gene Ontology (GO) terms. In addition, we investigated within-species phylogenetic structure for uSGBs using StrainPhlAn 4 (ref. ^25^) and evaluated the resulting 112 within-SGB phylogenies to assess differential strain carriage by CRC phenotypes and for subclade association with CRC-related microbial genes.

### CRC gut microbiome signatures are stage- and location-specific

**Extended Data Fig. 1:**
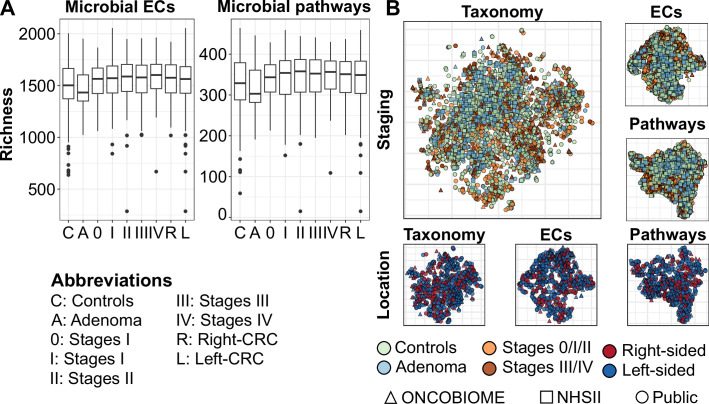
Alpha and beta-diversity analysis on taxonomic and functional profiles in CRC stages and primary tumor location. **a**) Richness of microbial enzymes and pathways in controls, adenomas, the different CRC stages and primary tumor locations. The abbreviations used in this plot follow the ones in Main Fig. 1. In particular, C: control, A: adenoma; 0: CRC Stage 0; I: CRC Stage I; II: CRC Stage II; III: CRC Stage III; IV: CRC Stage IV; R: right-sided CRC; L: left-sided CRC. **b**) t-SNE based on Bray-Curtis dissimilarity for taxonomic and functional profiles. The samples are colored according to CRC staging in the first row (light-green for controls, light-blue for adenomas, orange for early-CRC and brown for late-CRC), while they are colored according to primary tumor location in the second row (red for right-sided CRC and blue for left-sided CRC). The cohort derivation (either from ONCOBIOME, NHSII or public studies) is indicated as the shape of each point (triangle for ONCOBIOME, square for NHSII and a circle for public cohorts).

**Extended Data Fig. 2:**
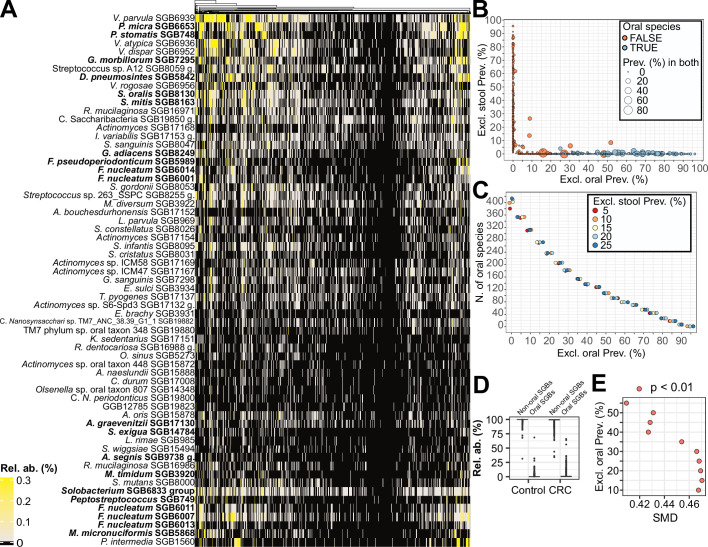
Oral SGBs abundance in CRC and evaluation of the definition criteria for the oral-to-gut score. **A**) CRC sample relative abundance (%) of highly prevalent oral species (at least 10% in CRC). Each column corresponds to a sample and each row corresponds to an SGB. The upper threshold in the heatmap color scale corresponds to the 99th percentile of oral species relative abundance in CRC samples. Relative abundance values higher than this threshold are represented with the same color. **B**) Exclusive and common prevalence of SGBs in the oral cavity and in the stool samples of the cohorts used for the definition of the oral SGB list and (**C**) trend in the number of SGBs in the list at different thresholds of exclusive oral prevalence and exclusive stool prevalence. **D**) Distribution of the cumulative relative abundances of species categorized as oral or non-oral. **E**) Meta-analysis effect sizes (Hedges’ g) of the oral-to-gut score computed based on different thresholds of the ‘Exclusive oral prevalence’.

Consistent with previous reports^18^, gut microbial alpha-diversity was higher in CRC than controls in 9 of 16 cohorts (SMD > 0, only two with *P* < 0.05), but this was not a particularly strong effect according to the meta-analytic approach via standardized mean differences (SMD), which was not statistically significant (*P* ≥ 0.05) (Fig. 1c,d, Extended Data Fig. 1a and Supplementary Table 4). We observed no clear relationship between richness and clinical stage compared with controls. Estimated oral-to-gut microbiome score (Methods and Extended Data Fig. 2a–e) was instead higher both in CRC cases (Hedges’ SMD = 0.47, *P* < 0.001) (Fig. 1e) and in later CRC stages (Hedges’ SMD = 0.14, *P* = 0.003). In addition, CRC originating from the right colon presented lower richness (Hedges’ SMD = 0.25, *P* = 0.07) (Fig. 1d and Supplementary Table 4) and a higher presence of orally derived SGBs than CRC originating from the left colon and rectum (Hedges’ SMD = −0.23, *P* = 0.003) (Fig. 1e and Supplementary Table 4).

Control and CRC microbiomes were clearly compositionally distinct, confirming previous findings (proportion of sum of squares *R*^2^ = 0.014, permutational multivariate analysis of variance (PERMANOVA) *P* ≤ 0.01) (Fig. 1f, Extended Data Fig. 1b and Supplementary Table 5). Stage 0–III microbiomes were not different from stage IV (*R*^2^ = 0.01, *P* ≥ 0.05), and stages 0–II (early) were not different from stages III–IV (late) (*R*^2^ = 0.01, Bray–Curtis; PERMANOVA *P* ≥ 0.05) (Fig. 1f and Supplementary Table 5). In addition, the microbiome of patients with adenoma did not differ significantly from controls (Fig. 1f and Supplementary Table 5), suggesting a more crucial role for the gut microbiome in the adenoma–carcinoma transition compared with earlier phases. Primary locations (right versus left) showed microbiome differences (*R*^2^ = 0.017, *P* = 0.002) (Fig. 1f and Supplementary Table 5) with no strain-level contribution to the separation (Fig. 1f and Methods). Altogether, the combined data support the potential of enriched oral microbial infiltration into the gut microbiome as a differentiator of CRC stages and locations (Fig. 1e,f).

### Improved CRC screening potential of gut metagenomics

**Fig. 2:**
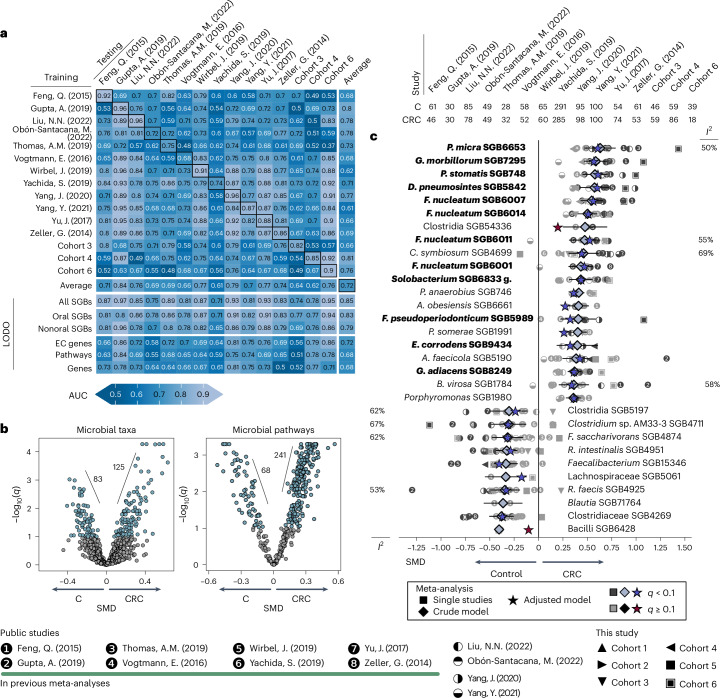
Stool microbiome composition is predictive for CRC. **a**, Cross-prediction matrix of the ML classifier trained and tested to predict CRC versus control samples. Dataset-wise CV AUCs are reported along the diagonal. Off-diagonal cells report performances of a classifier trained on the cohort on the row and tested on the cohort on the column. Bottom six rows report different LODO validations obtained by training a classifier on all the cohorts but one and testing on the left-out cohort (column), on the complete taxonomic profiles (All SGBs), the subset of oral SGBs (Oral SGBs), the subset of all SGBs except the oral SGBs (Nonoral SGBs), gene families clustered according to the EC numbers (EC genes) and MetaCyc pathways (Pathways), respectively, and a filtered set of gene families (Genes) (Methods). **b**, Significance levels of meta-analysis on microbial taxa and pathways between controls and CRC. The *x* axis shows the SMD and the *y* axis shows −log_10_(*q*). Positive values of Hedges’ *g* SMD indicate a positive association with CRC (higher abundance in CRC than controls), while negative values indicate a positive association with controls (higher abundance in controls than CRC). Associations with *q* < 0.1 are reported in blue. *I*^2^ values for heterogeneity in meta-analysis are reported if ≥50% **c**, Twenty strongest SGBs associated with CRC and ten with controls. Each line on the *y* axis reports the set of single-dataset and Hedges’ model SMD (values on the *x* axis, with the same directions as in **b**, represented by symbols and diamonds, respectively. Significant single-dataset comparisons (*q* < 0.1) are colored dark gray. Only significant Hedges’ *g* significant values (*q* < 0.1) are reported. SMD values corrected for age, sex and BMI (Methods) are indicated by a star (colored blue when *q* < 0.1). Oral SGBs are highlighted in bold. g, group.

ML applied to stool metagenomics can be a potential option for noninvasive CRC screening^18,19,28^. Here, we tested whether leveraging increased sample sizes and methods could further improve predictions of CRC cases. To do so, we exploited ML algorithms models^18,28,29^ in three different ways: (1) 10-fold cross-validation (CV) applied 20 times on each dataset separately (per-dataset CV); (2) a training–testing approach applied to pairs of distinct datasets (between-dataset CV); and (3) a leave-one-dataset-out (LODO) setting, in which the classifier was trained on all but one dataset and tested on the left-out dataset (iterated over each left-out dataset) (Methods and Fig. 2a).

Predictions of CRC status using a LODO approach achieved the highest and most stable area under the curve (AUC) values (average AUC = 0.85, ranging from 0.71 to 0.97) (Fig. 2a) and were an improvement compared with previous studies (average LODO AUC = 0.81)^18^. Predictions based on CV were, as expected, generally high but variable across datasets (average AUC ± s.d. = 0.87 ± 0.09, ranging from 0.68 to 0.96), with similar results for between-dataset CV (average AUC > 0.72 ± 0.11) (Fig. 2a).

We then tested the use of only oral and nonoral SGBs for CRC case versus control classification and obtained similar AUC values to the model considering all SGBs (average LODO AUC = 0.83 compared with 0.85 when considering only oral SGBs and 0.79 when considering nonoral SGBs) (Fig. 2a), confirming that a large part—but not all—of the predictive power of the microbiome for CRC lies in the presence of oral-typical taxa in the stool. By contrast, ML models using different sets of microbiome functional features were less predictive (average LODO AUC of 0.68 to 0.72). These results reinforce the potential of predictive tools applied to stool metagenomics to be useful for CRC screening when trained on large and diverse datasets and highlight the predictive importance of oral species present in the gut during CRC.

### Oral and newly associated SGBs enriched in the CRC microbiome

**Extended Data Fig. 3:**
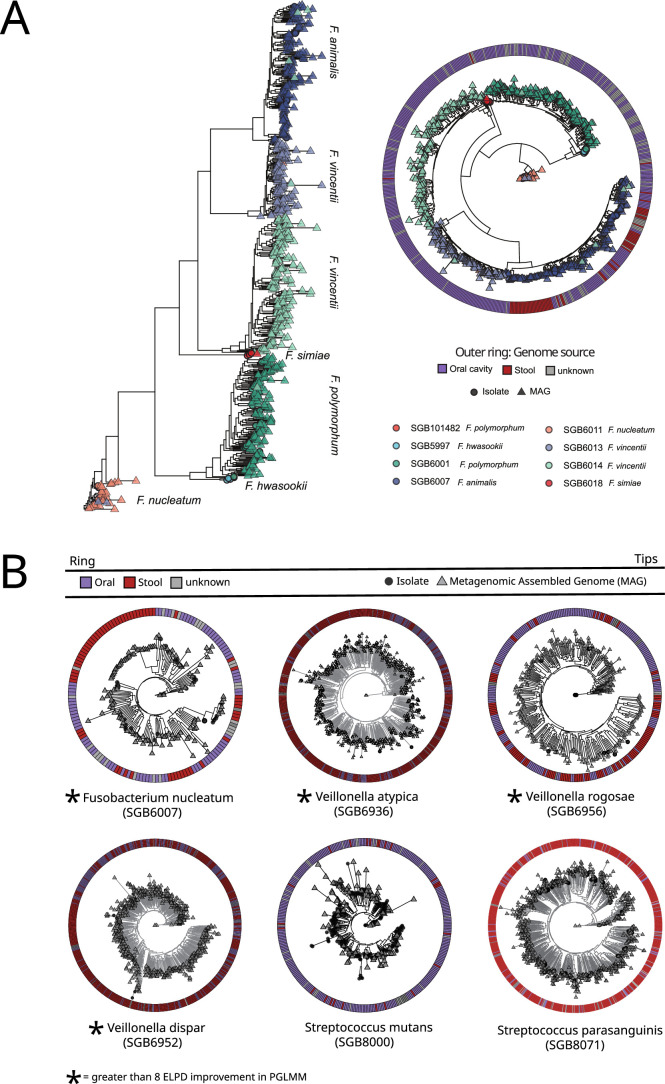
Phylogenetic trees of Fusobacterium nucleatum SGBs and CRC-associated species. **A**) The left-most phylogenetic tree shows the relationships between *F. nucleatum* SGBs and previously characterized *F. nucleatum* subspecies. Each leaf represents either an isolated genome (circle) or a metagenome-assembled genome (MAG, reported as a triangle), as available in the Jun23 release of the MetaPhlAn 4 database. The color of the leaf corresponds to the assigned SGB. Phylogenies were reconstructed using PhyloPhlAn 3. The correspondence between *F. nucleatum* SGBs and *F. nucleatum* subspecies is the following: subspecies *hwasookii* corresponds to SGB5997, subspecies polymorphum to SGB6001, subspecies *animalis* to SGB6007 (previously Fna C2, in 26), subspecies *nucleatum* to SGB6011 (*Fusobacterium nucleatum sensu stricto*), subspecies *vincentii* to both SGB6013 (previously Fna C1, in 26) and SGB6014, *Fusobacterium simiae* to SGB6018 and subspecies *polymorphum* to SGB101482. The right-most tree reports in addition the body site of origin from which the genomes were reconstructed. **B**) Phylogenetic reconstruction via PhyloPhlAn 3 of isolate and MAGs either derived from oral, stool, or unknown body site for *Fusobacterium nucleatum* SGB6007 (*F. nucleatum* subspecies *animalis*), *Veillonella atypica* SGB6936, *Veillonella rogosae* SGB6956, *Veillonella dispar* SGB6952, *Streptococcus mutans* SGB8000, and *Streptococcus parasanguinis* SGB8071. SGBs with ELPD > 8 from the PGLMM with body-site of origin as target variable are marked with an asterisk.

**Extended Data Fig. 4:**
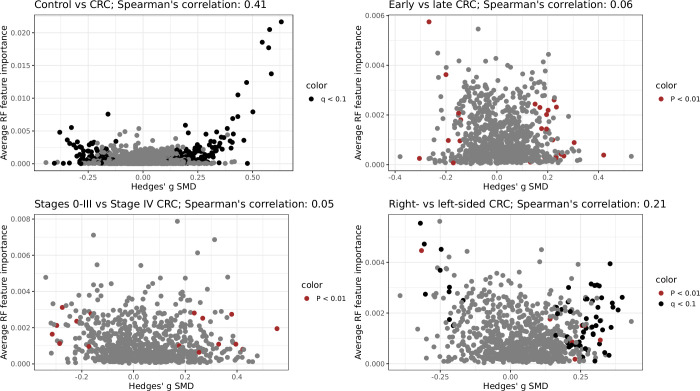
Correlation of the machine learning average ranks and the meta-analysis effect sizes of species in the comparisons of the study. Correlation of the effect sizes estimated via meta-analysis (Hedges’ g SMD values, on the x-axis) with the average ranked feature importance derived from the machine learning (LODO, on the y-axis). Significant SGBs detected via the meta-analysis for the comparisons tested in the manuscript (controls vs CRC, early stages CRC vs late stages CRC, non-metastatic vs stage IV CRC and right-sided vs left-sided CRC) are in black (q < 0.1) and red (P < 0.01). The comparison controls vs CRC showed the highest Spearman’s correlation coefficient (0.41), with right-CRC vs left-CRC Spearman’s correlation coefficient of 0.21. This confirms the stronger biomarkers obtained by these comparisons, while the comparisons on stages presented non-significant correlation coefficients (Spearman’s correlation coefficient of 0.06 and 0.05 for early-stages vs late-stages and non-metastatic vs metastatic CRC, respectively).

We next aimed to pinpoint specific microbiome biomarkers associated with CRC using the increased power of our multicohort framework (Methods). We identified 125 SGBs with increases relative abundance in CRC (*q* < 0.1, 106 kSGBs and 19 uSGBs) and 83 SGBs more abundant in controls (53 kSGBs and 30 uSGBs) (Fig. 2b and Supplementary Table 6); none of the eukaryotic species were differentially abundant (Supplementary Table 7). Bacterial biomarkers for CRC encompassed not only known associations, such as *Parvimonas micra*, *Gemella morbillorum* and *Peptostreptococcus stomatis* (SMD = 0.63, 0.59 and 0.58, respectively)^18^, but also newly associated SGBs such as multiple genomically distinct *F. nucleatum* SGBs (SGB6007 (*Fna* C2 in ref. ^23^), *F. nucleatum animalis*, SGB6014 *F. nucleatum vincentii*, SGB6011 *F. nucleatum* subsp. *nucleatum* (*F. nucleatum sensu stricto*), SGB6001 *F. nucleatum polymorphum*, SGB6013 (*Fna* C1 in ref. ^23^) *F. nucleatum vincentii*; SMD = 0.54, 0.5, 0.47, 0.43, and 0.34, respectively) (Extended Data Fig. 3a and Supplementary Table 6), two *Bacteroides fragilis* SGBs (SGB1853 and SGB1855 group; SMD = 0.32, *q* < 0.1) and two *Hungatella hathewayi* SGBs (SGB4741 and SGB4742; SMD = 0.33 and 0.27, *q* < 0.1) (Fig. 2c and Supplementary Table 6). Of the 19 associated uSGBs, two (SGB63163 and SGB63167) were assigned at the phylum level (Firmicutes), four (SGB3996, SGB4367, SGB14306 and SGB14315) at the class level (Clostridia) and nine at the family level. Only four SGBs were uncharacterized at the species level (that is, belonging to known genera): *Solobacterium* SGB6833 (distinct form the previously associated *Solobacterium moorei*^18^), *Peptostreptococcus* SGB749 and two *Porphyromonas* SGBs. The effect sizes of the signature identified via meta-analysis significantly correlated with the average ranks of the LODO-evaluated ML models (Spearman’s correlation coefficient = 0.41, *P* < 0.001) (Extended Data Fig. 4), thus supporting the robustness and consistency of the two independent approaches.

**Extended Data Fig. 5:**
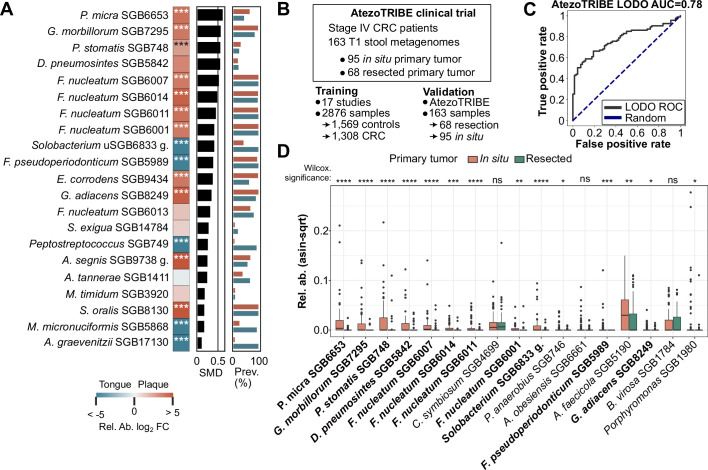
Differential abundance analysis between tongue dorsum and dental plaque of CRC associated oral species in the gut and validation of the CRC biomarkers in patients with *in situ* or resected primary tumors. **A**) Differential abundant oral SGBs in the signature of CRC between the oral plaque and tongue dorsum in the Human Microbiome Project (HMP) study. We selected 86 participants of the HMP study with matched microbiome samples for the two oral cavity sites. Differential abundance analysis was performed via Wilcoxon signed-rank test and adjusted p-values (q) were computed via Benjamini-Hochberg (BH) correction. q < 0.001 is indicated with ‘***’. log2 fold-change (FC) between average relative abundances (RA) in the two sites is reported in the heatmap. Positive values of the RA log2 FC indicate increased relative abundance in the plaque, while negative in the tongue dorsum subsite. **B-D**) Validation of the machine learning and CRC gut microbiome signature between patients with in situ or resected primary tumor in the AtezoTRIBE study.

A considerable fraction of the identified CRC gut biomarkers were oral-typical species: 21 of the 125 SGBs (16.8%) positively associated with CRC were oral-typical, in contrast to 34 of 488 (7.0%) nonsignificantly associated with CRC (Fisher’s test, *P* < 0.01). No oral SGB was associated with controls and a greater proportion of oral SGBs were associated with CRC at lower *q* thresholds (18 of 90 at *q* < 0.05). By reconstructing strain- and subspecies-level phylogenies for oral-typical species associated with CRC via PhyloPhlAn 3 (ref. ^30^) using metagenome-assembled genomes and isolates, we further identified several subclades of taxa that appear to be more prevalent in the oral cavity or the gut, including clades of *F. nucleatum* SGB6007 and three *Veillonella* species (Extended Data Fig. 3b). Within oral species, there is thus evidence of genomic adaptation to the intestinal environment. In addition, to better characterize the tropism of the 21 oral SGBs that are more abundant in CRC (Extended Data Fig. 5a), we exploited datasets with dental plaque and tongue dorsum metagenomes from the same individuals^31^. We determined that 11 SGBs were more abundant in the dental plaque (7 of the top 8 species), whereas 5 were more abundant in the tongue dorsum (Extended Data Fig. 5a), thus hinting at a potential major contribution of biofilm-forming microbes in the intestinal CRC microbiome.

To test whether the microbiome biomarkers for CRC were associated with the presence of the primary tumor in the gut, we evaluated the microbiome potential to discriminate between patients with stage IV CRC who had an in situ primary tumor and patients with resected primary tumor from the AtezoTRIBE study. We obtained a LODO AUC = 0.78 with a classifier trained on all the other studies for distinguishing between cases and controls. In addition, 13 (11 oral) of the 20 SGBs most associated with CRC (Fig. 2b) were also significantly (*P* < 0.05) more abundant when the primary tumor is present rather than resected (Extended Data Fig. 5b–d). Overall, this reinforces the relevance of oral-to-gut introgression by oral commensals^18^ and that the primary tumor microenvironment determines the overall stool microbiome signature in CRC.

### Associations between CRC and functional microbiome profiles

**Extended Data Fig. 6:**
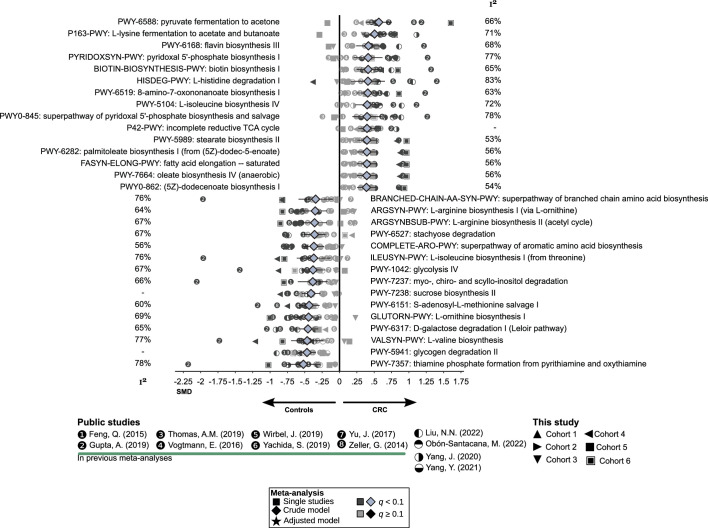
Microbial pathways strongest associated either with CRC or controls. Differentially abundant microbial pathways (meta-analysis model q < 0.1) between controls and CRC. Associations with CRC present Hedges’ g SMD > 0, and associations with controls present Hedges’ g SMD < 0. Significant single-dataset comparison (q < 0.1) is reported in dark gray, while non-significant single-dataset associations (q ≥ 0.1) is in lighter gray.

**Extended Data Fig. 7:**
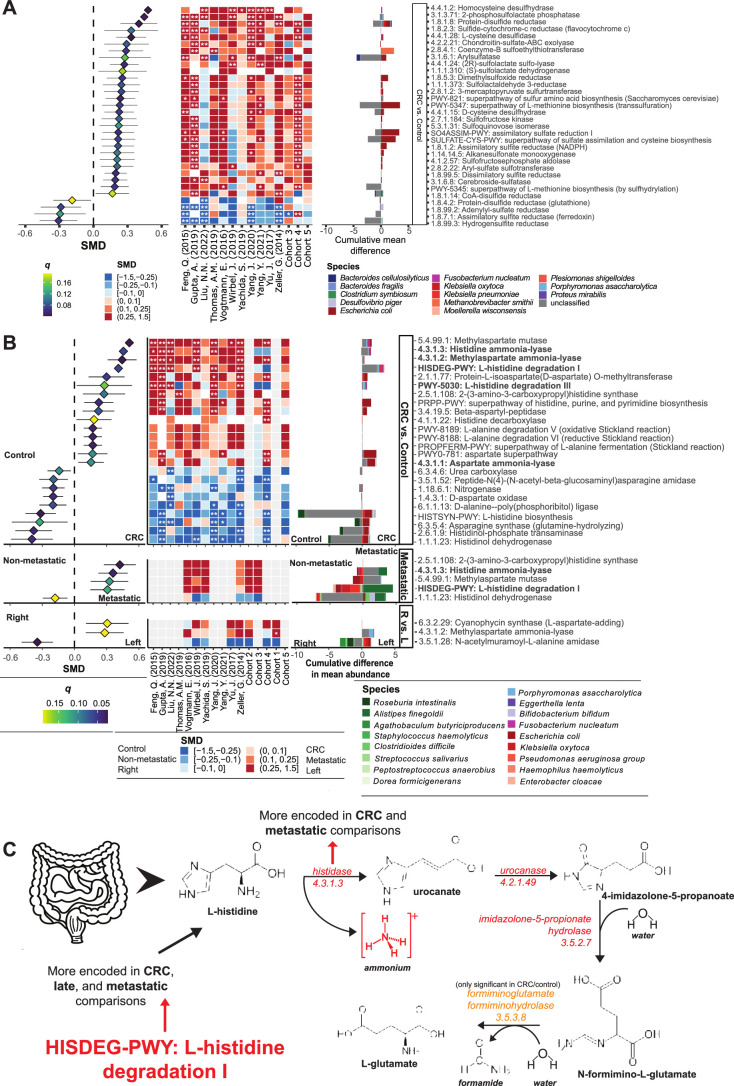
Associations of sulfur-related and histidine related pathways with CRC, tumor stages and primary tumor location. **A**) Meta-analysis of sulfur-related pathways for controls vs CRC. Hedges’ g meta-analysis SMD and 95% confidence intervals are reported in the first plot. Significance level is indicated via a color gradient. P-values have been adjusted via Benjamini-Hochberg procedure for controlling FDR. Single cohort effect size levels are reported in the heatmap, ranging from dark blue for SMD = −1.5 to dark red for SMD = 1.5. Mean difference in abundance with species contribution for each pathway is reported in the stacked barplot on the right. **B-C**) Overview of histidine-related microbiome pathways and their associations with CRC, staging and primary tumor location. **B**) Meta-analysis of histidine-related pathways for controls vs CRC, early CRC (Stages 0-II) vs late CRC (Stages III-IV), non-metastatic CRC (Stages 0-III) vs metastatic CRC (Stage IV), and right-sided CRC and left-sided CRC. Hedges’ g meta-analysis SMD and 95% confidence intervals are reported in the first plot. Significance level is indicated via a color gradient. P-values have been adjusted via Benjamini-Hochberg procedure for controlling FDR. Single cohort effect size levels are reported in the heatmap, ranging from dark blue for SMD = −1.5 to dark red for SMD = 1.5. Mean difference in abundance with species contribution for each pathway is reported in the stacked barplot on the right. C) Visual representation of the biochemical mechanisms that lead to L-glutamate production from L-histidine, with the production as a side product of ammonium.

We investigated the microbiome’s functional repertoire alteration in CRC-affected individuals (Supplementary Table 6). In agreement with previous work^18^, the *cutC* gene showed higher abundance in CRC-associated metagenomes (CRC versus control SMD = 0.28; *q* = 0.001) (Supplementary Table 6). In total, 241 MetaCyc pathways were also positively associated with CRC (*q* < 0.1) and 68 with controls (Fig. 2b, Extended Data Fig. 6 and Supplementary Table 6). At the enzyme level, sulfur-producing enzymes were associated with CRC (including EC 4.4.1.2 homocysteine desulfhydrase (SMD = 0.52, *q* < 0.001) and EC 1.8.1.8 protein disulfide reductase (SMD = 0.41, *q* < 0.001)), consistent with previous work^32^ (Extended Data Fig. 7a and Supplementary Table 6). In addition, the association between CRC and two pathways involved in the production of oleate in aerobes (PWY-6282 (SMD = 0.4, *q* < 0.1) and PWY-7664 (SMD = 0.9, *q* < 0.1)) (Extended Data Fig. 6, Supplementary Table 6) corroborated a previous hypothesis of an association between oleate and the proliferation of cancerous cells^33^.

We then tested whether increased ammonia levels are characteristic of CRC tumor microenvironments^34^ and found several pathways and enzymes involved in ammonia production or sequestration that were significantly altered in the presence of CRC. In particular, l-histidine degradation pathways were more frequently encoded in CRC metagenomes (meta-analysis SMD = 0.41, 0.32, respectively, *q* < 0.1) (Supplementary Table 6). The first step of this pathway involves the enzyme histidase (EC 4.3.1.3) cleaving an amino group off l-histidine to create urocanate and ammonia as by-products, and this histidase was similarly enriched in CRC (SMD = 0.46, *q* < 0.1), late CRC (SMD = 0.18, *P* = 0.02) and metastatic CRC metagenomes (SMD = 0.35, *q* < 0.1). In addition, a second ammonia lyase enzyme, methylaspartate ammonia lyase (EC 4.3.1.2), was also highly associated with CRC metagenomes (SMD = 0.45, *q* < 0.1) and the opposite for l-histidine biosynthesis (SMD = −0.33, *q* < 0.1) (Extended Data Fig. 7b,c and Supplementary Table 6). The tumor microenvironment of CRC was previously characterized as having increased levels of host-produced ammonia, leading to T cell exhaustion^34^, but our results suggest a potential role for gut microbiota in ammonia regulation in the tumor microenvironment.

### CRC stages display partially different stool microbiomes

**Fig. 3:**
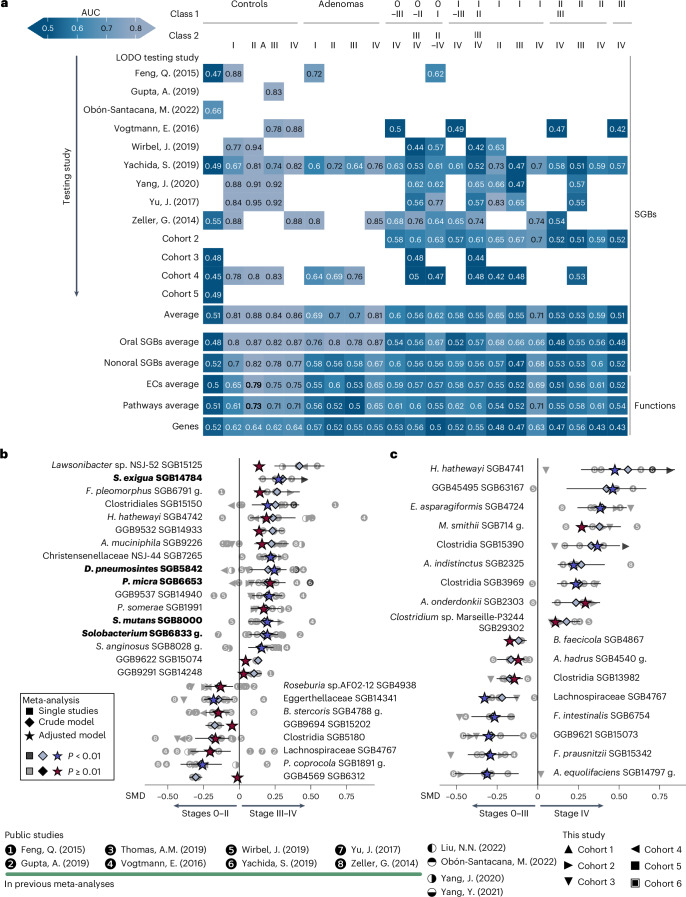
CRC stage prediction and microbiome signatures for early and late stages of CRC. **a**, LODO AUC predictions based on taxonomic microbiome composition (upper) and microbial functional potential (lower). Paired comparisons are indicated in the header (Class 1 versus Class 2). Each cell contains the AUC from a random forest validated in LODO, tested on the study reported in the row and trained on the remaining studies. The last five rows of the matrix report the average LODO AUC values predicted using oral microbes, all SGBs except the oral ones, EC profiles, pathways profiles and a subset of the gene families (Methods). **b**,**c**, Significant species (Hedges’ model effect size *P* < 0.01, none presented *q* < 0.1) found in association either with earlier or later stages in meta-analysis considering the following comparisons (all presented *I*^2^ < 50%). **b**, Stages 0–II versus stages III–IV. **c**, Stages 0–III versus stage IV. The shape of each point indicates the dataset-specific effect size for each species, and the blue diamonds indicate Hedges’ model effect size on SMD. *P* values were computed via two-tailed *t*-test. SMD values were corrected for age, sex and BMI and indicated by a star (blue if *P* < 0.01). Oral SGBs are highlighted in bold. g, group.

To assess whether CRC-associated gut microbiome alterations are stage specific, we considered samples in each stage separately as well as combined into early (0–II) versus late (III–IV) CRC. We observed no strong differences when discriminating between healthy individuals and patients with colorectal adenoma (Fig. 3a). By contrast, controls and adenomas were distinct when contrasted to early or late CRC stage metagenomes (Fig. 3a) with the highest AUC values obtained when comparing stage II and stage IV against controls (average AUC of 0.88 and 0.86, respectively) and adenomas (average AUC of 0.7 and 0.81) (Fig. 3a). The high AUC values obtained when classifying controls versus early stages individually confirm that the microbiome already differentiates at the beginning of the disease. Single stages were also partly distinguishable among themselves (AUC of 0.65 and 0.71 for stage I versus stage II and for stage I versus stage IV, respectively) (Fig. 3a).

We then investigated which microbial SGBs were differentially abundant in early and late CRC stages, as well as in metastatic CRC. We found 17 SGBs associated with late CRC, but only four associated with early CRC (Fig. 3b and Supplementary Table 6). Among the former were five SGBs of oral origin: *Slackia exigua* SGB14784, *P. micra* SGB6653, *Solobacterium* SGB6833, *Dialister pneumosintes* SGB5842 and *Streptococcus mutans* SGB8000. Interestingly, *P. micra* SGB6653 was already increased in stage I, along with *G. morbillorum* SGB7295, while *F. nucleatum* SGB6007, despite being significantly increased in stage I, appeared to be consistently more abundant starting in stage II of CRC (Supplementary Table 8). We note that stage-specific taxa are usually part of the whole CRC versus healthy state signature (8 of the 17 significant SGBs between early versus late CRC) indicating a continuum in microbiome trends along stages rather than distinct configurations.

Among the nine SGBs significantly more abundant in stage IV, *H. hathewayi* SGB4741 showed the greatest increase in abundance (Fig. 3c) with *Methanobrevibacter smithii* SGB714 also among the top associated species (Fig. 3c and Supplementary Table 6).

**Extended Data Fig. 8:**
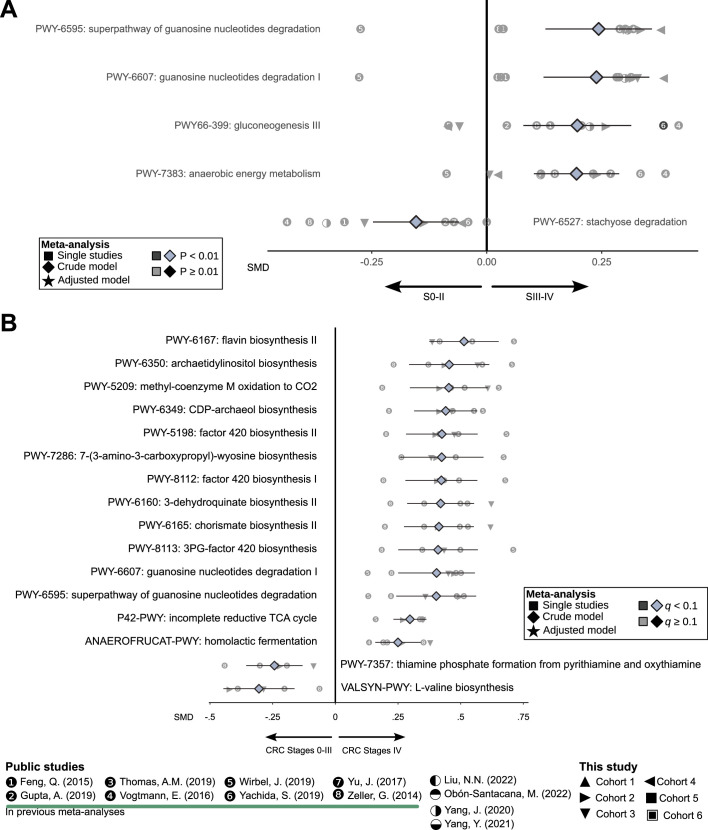
Microbial pathways that are differentially abundant in meta-analysis between early- vs late CRC and non-metastatic vs metastatic CRC. **A**) Differentially abundant microbial pathways (meta-analysis model P < 0.01) between early (stages 0-II) and late (stages III-IV) CRC. Associations with CRC Stage III-IV present Hedges’ g SMD > 0, and associations with CRC Stages 0-II present Hedges’ SMD < 0. Significant single-dataset comparison (P < 0.01) is reported in dark gray, while non-significant single-dataset associations (P ≥ 0.01) is in lighter gray. **B**) Differentially abundant microbial pathways (meta-analysis model q < 0.1) between non-metastatic (stages 0-III) and metastatic (stage IV) CRC. Associations with stage IV present SMD > 0, and associations with CRC Stages 0-III present SMD < 0. Significant single-dataset comparison (q < 0.1) is marked in dark gray, while non-significant single-dataset associations (q ≥ 0.1) are in lighter gray.

Considering pathways, we found four pathways differential between early versus late CRC (*P* < 0.01) (Extended Data Fig. 8a and Supplementary Table 6), while 14 were increased in stage IV versus all the other stages combined (*q* < 0.1) (Extended Data Fig. 8b). Although few microbial pathways were found to be associated with late or early CRC stages, we confirmed the previously reported association between methane metabolism and stage IV CRC^15^ (METHANOGENESIS-PWY, SMD = 0.4, *P* < 0.01) (Supplementary Table 6).

### Consistent SGB trends along CRC stages

**Fig. 4:**
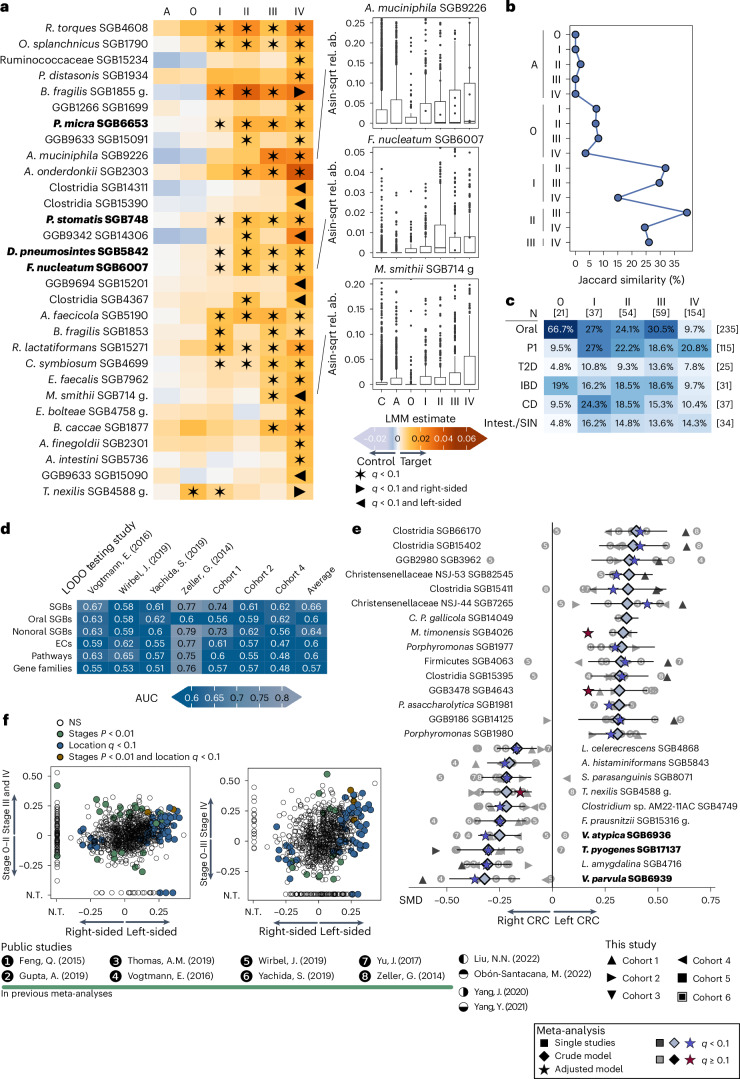
Stage-specific microbial signatures overlap with oral and cardiometabolic risk signatures and microbiome differences in right- and left-sided tumors. **a**, Linear mixed model coefficients (in the heatmap cells) showing the associations between each microbial species and each stage when compared with controls. Positive values (from orange to brown) indicate increased stage SGB abundances compared with in controls, while negative coefficients (blue) indicate decreased abundances. Significant associations (*q* < 0.1) are indicated by a star. Associations also found significant in either right- or left-sided CRC for each stage (*q* < 0.1) are indicated by a right- or left-pointing triangle, respectively. Oral SGBs are highlighted in bold. Box plots represent the distribution of three SGBs with significant changes in the abundances in CRC stages. **b**, Jaccard similarities between the signatures of sequential CRC stages. **c**, Overlap for CRC stages signatures with oral SGBs and the species associated with cardiometabolic risk in the PREDICT 1 (P1) study, with T2D, IBD, CD and inflammatory diseases (Intest./Syst. infl.). The number of stage-associated SGBs is reported on top of each column. The number of SGBs in each signature is reported at the end of each row. **d**, LODO AUC for right-sided versus left-sided CRC classification, considering all SGBs, oral SGBs only, nonoral SGBs, EC numbers, MetaCyc pathways or a subset of all the gene families (Methods). **e**, SGBs significantly associated (*q* < 0.1) either with right- or left-sided CRC. Meta-analysis Hedges’ *g* is indicated by a diamond, while the SMD values corrected for age, sex and BMI are indicated by a star (blue if *q* < 0.1). Oral SGBs are in shown bold. **f**, SMD values of a meta-analysis of right- versus left-sided tumor-related microbiome composition (*y* axis) versus the coefficients of a meta-analysis of stages 0–II versus stages III–IV (*x* axis, left), and 0–III versus stage IV (*x* axis, right) for taxonomic profiles. Common signature SGBs are in shown orange (*q* < 0.1 and *P* < 0.01, respectively); SGBs significant in the cancer stages meta-analysis are shown in green (*P* < 0.01); SGBs significant in the meta-analysis for tumor location are shown in blue (*q* < 0.1); and SGBs not significant in both analyses are shown in gray. Asin-sqrt rel. ab., arcsine square root transformed relative abundance; C, *Candidatus*; g, group; Intest., ; N.T., not tested in the corresponding analysis.

**Extended Data Fig. 9:**
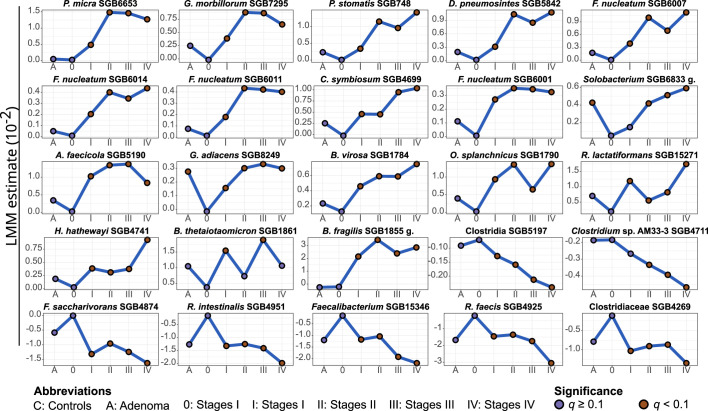
SGBs strongest associated either with CRC or controls present different relative abundance changes along CRC stages. Each line-plot represents the trends of an SGB in adenoma and the different stages of CRC in relation to controls’ abundances. Each point in the line is the coefficient from the linear mixed model (MaAsLin2) run to determine the association for each SGB and the different stages. Non-significant coefficients (q ≥ 0.1) are reported with a violet circle, while significant coefficients (either positive or negative, q < 0.1) are reported as a brown diamond. The x-axis of each line plot indicate the control, adenoma or stage: C for control, A for adenoma; 0 for CRC Stage 0; I for CRC Stage I; II for CRC Stage II; III for CRC Stage III; IV for CRC Stage IV.

We then investigated SGBs showing particularly consistent trends of increased or decreased abundance across all CRC stages (Methods, Fig. 4a and Extended Data Fig. 9). Two such examples were *P. micra* SGB6653 and *F. nucleatum* SGB6007, in which the abundance started to increase at stage I (Fig. 4a and Extended Data Fig. 9). Conversely, *Akkermansia muciniphila* (SGB9226 and SGB9228) and *Parabacteroides distasonis* SGB1934 were generally more abundant in later stages of CRC (Fig. 4a and Extended Data Fig. 9). These results suggest that the microbiome changes in late-stage CRC^17–19^ occur predominantly on a continuum and become more pronounced as the cancer progresses (Fig. 4b).

Because cardiometabolic disorders and CRC share many risk factors^35^, we quantified the overlap in microbial biomarkers between adenoma and CRC stages with respect to oral species, other human diseases and cardiometabolic markers^36^ (Fig. 4c and Methods). SGBs characteristic of CRC stages I–IV included ~25% of species associated with cardiometabolic risk; stage 0 CRC shared the highest proportion of oral bacteria compared with the other stages (58%); stage I CRC showed the highest percentage of species in common with poor cardiometabolic health (27%) (Fig. 4c). All CRC stages shared SGBs with Crohn’s disease (CD) and immune-mediated diseases (Fig. 4c), while stage IV shared 21% of SGBs with poor cardiometabolic health markers and less with immune-mediated diseases (Fig. 4c). Importantly, the microbial signatures of these three conditions have only two SGBs in common (*H. hathewayi* SGB4741 and *Enterocloster aldensis* SGB476). Overall, these results indicate a high proportion of SGBs associated with poor cardiometabolic health in all stages of CRC and in adenomas, and a high degree of oral species during CRC development, which is also shared across inflammatory diseases (intestinal or systemic)^37,38^, and generalized inflammatory conditions characterizing metastatic tumors.

### Gut microbiomes differ according to primary tumor location

We found that differences in the mucosal microbiome according to primary tumor location^21,22^ extend to stool metagenomics (average AUC = 0.66 across cohorts) when using all SGBs (min = 0.58, max = 0.77) and similarly when limited to oral SGBs (average AUC = 0.6) (Fig. 4d). This underscores a difference in microbiome composition that can be relevant for side-specific mechanistic models.

**Extended Data Fig. 10:**
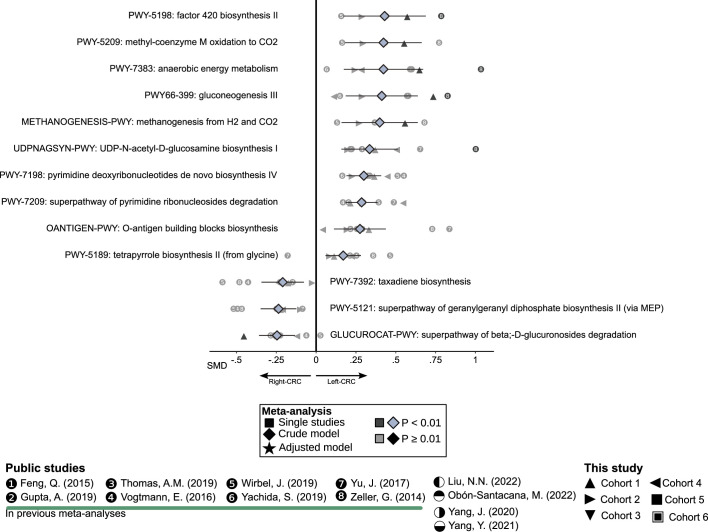
Microbial pathways that are differentially abundant between right-sided and left-sided CRC. Differentially abundant microbial pathways (meta-analysis model P < 0.01) between right-sided and left-sided CRC. Pathways associated with right-sided CRC present SMD < 0, and pathways associated with left-sided CRC present SMD > 0. Significant single-dataset comparisons (P < 0.01) are marked in dark gray, while non-significant single-dataset associations (P ≥ 0.01) are in lighter gray.

Among the 61 tumor location differential SGBs (*q* < 0.1), we found that three oral-typical SGBs (*Veillonella parvula* SGB6939, *Veillonella atypica* SGB6936 and *Trueperella pyogenes* SGB17137) were significantly increased in right-sided CRC (Fig. 4e Supplementary Table 6). In addition, seven of the ten SGBs associated with right-sided CRC (*q* < 0.1) were nonoral SGBs. Among these, we identified *Streptococcus parasanguinis* SGB8071, which was shown to form biofilms with *Veillonella* spp.^39^, suggesting that such interactions may be more characteristic of right-sided CRC^22^. Importantly, only a few SGBs were found in common when comparing the signature for primary tumor location with CRC stages: Christensenellaceae NSJ_44 SGB7265 and *P. micra* SGB6653 when considering early versus late stages (Fig. 4f), and Clostridia SGB15390, *Clostridium* sp. Marseille P3244 SGB29302 and GB45495 SGB63167 when considering nonmetastatic versus metastatic CRC (Fig. 4f). We observed a similar behavior also when considering microbial pathways (Extended Data Figs. 8 and 10), reinforcing the notion that primary tumor location is a subtle but detectable factor to account for when studying gut microbiome alterations in patients with CRC.

### Strain differences in gene carriage during CRC

**Fig. 5:**
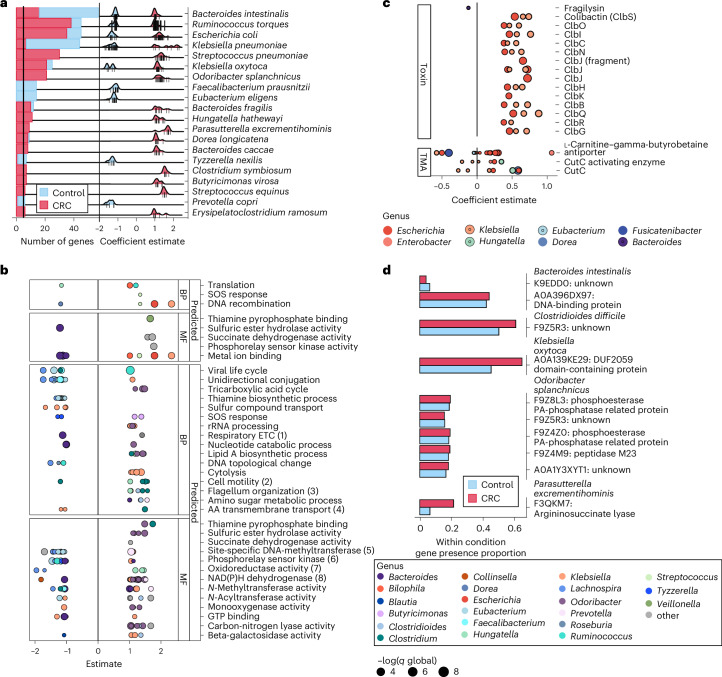
Strain-specific differential gene carriage in the CRC gut ecosystems. **a**, Top 20 species by the number of significantly differentially carried genes among their strains in CRC (FDR global *q* < 0.05 and absolute GLM coefficient estimate >1), likely representing species diversity. For the top 20 species, we quantified the number of genes they were differentially carrying along with the dispersion of such genes in CRC and healthy subjects as quantified by Anpan. Tick marks indicate individual genes. **b**, GO terms identified as having the highest ratio of significantly called UniRef90s (genes) in CRC to total genes defined in the term. GO terms are drawn from biological processes (BP) and molecular functions (MF) and are split across known gene families (annotated directly to GO by HUMAnN and predicted from FUGAsseM, https://huttenhower.sph.harvard.edu/fugassem/) using samples with paired metagenomic and metatranscriptomic data to assess the likely function of undescribed gene families. (1) Respiratory electron transport chain; (2) bacterial-type flagellum-dependent cell motility; (3) bacterial-type flagellum organization; (4) amino acid transmembrane transport; (5) site-specific DNA-methyltransferase (adenine-specific) activity; (6) phosphorelay sensor kinase activity; (7) oxidoreductase activity, acting on other nitrogenous compounds as donors; and (8) NAD(P)H dehydrogenase (quinone) activity. **c**, Carriage of genes previously known to associate broadly with CRC; colibactin, fragilysin and *cutC* genes, by specific clades’ strains in the CRC ecosystem as quantified by Anpan’s gene model. Although these genes did not show significance when assessed in species, they did at the global level (for example, when considering total carriage of the function in CRC metagenomes). **d**, The significant genes involved in the carbon–nitrogen GO term (**b**) were expanded and broken down by species carrying the genes. For each species-gene pair, we quantified the prevalence of the gene’s carriage in healthy controls and CRC cases. Many of the genes predicted to be in this category were annotated by FUGAsseM and as such are predictions of the potential function but are otherwise genes of unknown function. AA, amino acid; ETC, electron transport chain; rRNA, ribosomal RNA; TMA, trimethylamine.

We then investigated within-species differential gene carriage and diverging phylogenetic features among strains associated with CRC status and stage. We assessed differential gene family carriage (UniRef90s) for 179 SGBs detectable at sufficient prevalence using generalized linear models (GLMs). This identified 62 species that are typically not differentially abundant between cancer conditions, but rather whose strains differentially carried at least one gene family (UR90) in CRC (GLM false discovery rate (FDR) global *q* < 0.05 and absolute coefficient estimate >1) (Methods and Supplementary Table 9). Many of the species containing the most genetically differential strains were also identified as nonoral CRC-associated, including species of the genus *Klebsiella*, *E. coli*, *B. fragilis* and *H. hathewayi* (Fig. 5a). Nine of the 20 species with the highest number of differentially carried genes had genes enriched in both CRC and controls, highlighting the degree to which strains in the same SGB can differ in functional potential during CRC. Intriguingly, several species carrying a subset of genes solely in CRC were more typically quantified as human commensals, such as *Odoribacter splanchnicus* and *Dorea longicatena*, suggesting that some strains of these species may be unusually detrimental or CRC-responsive.

We then identified pathways specific to CRC-enriched or CRC-depleted strains using a subset of these differential genes that possessed at least some functional characterization. Many molecular functions were altered in CRC-associated microbes. Among the known functions identified using HUMAnN^28^ (Methods), the SOS response (GO:0009432)—a broad term for cellular response to DNA damage—was more frequently carried by *Streptococcus equinus* strains in CRC. Gene families from *Tyzzerella nexilis* and *Butyricimonas virosa* were also predicted to fall in this GO term (Methods) and be differentially carried in CRC. Consistent with community-wide results, we also found that succinate dehydrogenase activity was more encoded by *Parasutterella excrementihominis* strains in CRC, which also fits the hypothesis that this fatty acid is more readily available in the CRC-associated ecosystem^40^ (Fig. 5b).

Although not significant, the carriage of colibactin-producing genes by *E. coli* and *Klebsiella* spp. was increased in CRC (Anpan GLM; *q* > 0.01 and abs(coefficient estimate) < 1 (a measure of effect size)). This could indicate several potential hypotheses including: (1) that we did not capture the correct time point for an impact of *pks*^+^ *E coli* on CRC progression; (2) low levels of this gene are always encoded and activation is required for the toxicity^41^; or (3) colibactin is responsible for a minority of CRCs (Fig. 5c). We propose similar hypotheses for the *B. fragilis* toxin fragilysin, for which the gene was not enriched in CRC (Fig. 5c). Finally, no significant enrichment of cutC-related enzymes was observed, suggesting that the increased prevalence of this gene was due to the increased abundance of the species carrying this gene, and not the selection for this gene in a species (Fig. 5c).

Carbon–nitrogen lyase carriage was also of interest (Fig. 5b,d), because genes in this molecular function produce ammonia. Increased ammonia levels have been shown to contribute to T cell exhaustion and suppressed immune activity in CRC, and recently the microbiome was potentially implicated in this process in mice^34^. Here, we identified five species encoding genes involved in ammonia production in the CRC ecosystem, including *Klebsiella oxytoca*, *O. splanchnicus*, *Bacteroides intestinalis*, *P. excrementihominis* and *Clostridioides difficile* (Fig. 5d). These included the argininosuccinate lyase gene, which was carried by *P. excrementihominis* (Fig. 5d) and has ammonia as a known product of its molecular activity^34^.

### Within-species microbial subclades associate with CRC

**Fig. 6:**
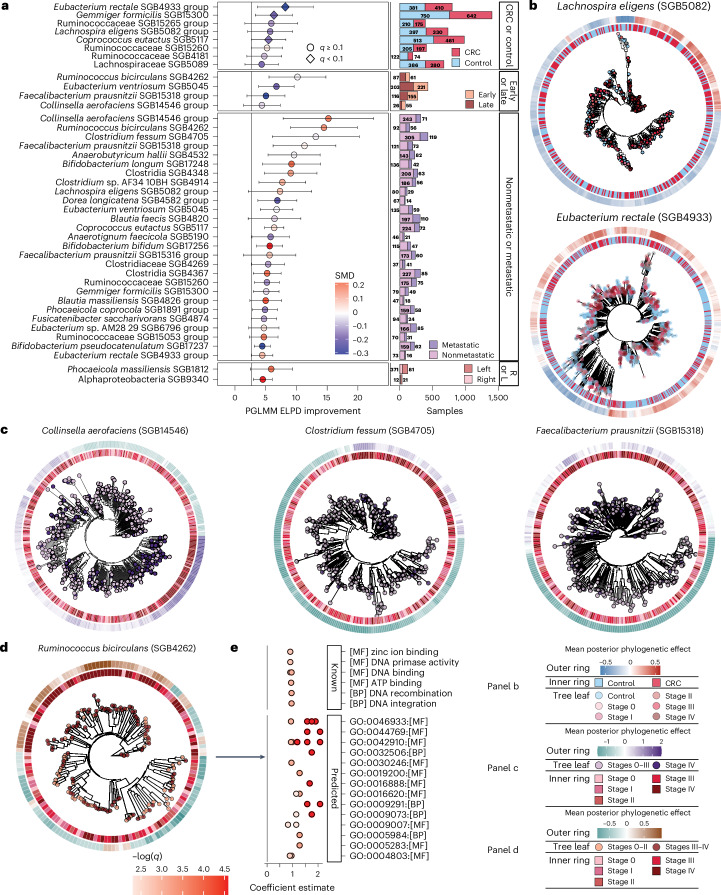
Within-species subclades associations with CRC, tumor staging and primary location. **a**, Several species exhibited within-species subclade associations with CRC, late CRC, metastatic CRC and the primary tumor location (right or transverse colon versus left colon or rectum). Only two of the associations were identified in species that were themselves differentially abundant with CRC in the meta-analysis, emphasizing the different pressures on colonization and growth versus phylogeny and evolution. Species are shown if the GLM improvement with phylogeny was greater than an ELPD of 4. Points are colored by the Hedges’ *g* value of the species-level meta-analysis in the given comparison, and the number of tips in the analysis is presented in the barplot, colored by CRC or control, early or late stages of CRC or right- or left-sided. Error bars represent 95% CI. **b**, Within-species subclade clustering obtained via Anpan analysis with CRC as the outcome of interest. Here, we highlighted two examples of *Lachnospira eligens* and *E. rectale* (full model results from Anpan in Supplementary Table 10) exhibiting phylogenetically distinct subclades associated with CRC or healthy individuals. For each cladogram, the inner ring is colored by CRC or control, tips are colored by stage and the outer ring is the mean posterior phylogenetic effect as calculated by Anpan’s phylogenetic generalized linear mixed model (PGLMM) model with covariates of age, sex and study. **c**,**d**, Within-CRC comparisons had more significant hits than global analysis (CRC or control). Here, we present some of the top hits from the model with *Collinsella aerofaciens* SGB14546, *Clostridium fessum* SGB4705 and *F. prausnitzii* 15318 (**c**) for the metastatic comparisons, and *R. bicirculans* SGB4262 (**d**) in late stages. Similar to **b**, the inner ring is the CRC stage, while tips are metastatic status and early or late, respectively, and the outer ring is the mean posterior phylogenetic effect (full model results from Anpan are given in Supplementary Table 10). **e**, Significant gene carriage differences by taxon associations in *R. bicirculans* (Anpan, *q* < 0.05 and abs(estimate) > 1). Genes differentially carried by *R. bicirculans* included those in carbohydrate metabolism, DNA mobility, and response to oxidative environments; all genes were found to be more present in late-stage CRC (III–IV) than in early-stage CRC (0–III).

We then expanded the gene carriage model that indicated the likelihood of distinct strain carriage in species in the CRC ecosystem, with a complementary within-species phylogenetic model via dominant strain profiling at single-nucleotide resolution using StrainPhlAn 4 (ref. ^25^). This identified several species with an expected log point-wise predictive density (ELPD) of 4, thus carrying dominant strains in distinct phylogenetic lineages in CRC (Fig. 6a and Supplementary Table 10), including early–late (Supplementary Table 10) and nonmetastatic–metastatic comparisons (Supplementary Table 10). We considered a clade significant if the phylogenetic model improved ELPD over a base GLM (the same model without phylogenetic information) by more than a factor of 2. Eight species exhibited differential strain carriage in the broad definition of CRC (stages 0–IV) (Fig. 6a, Supplementary Table 10 and Methods), while only two species were associated with primary tumor location (Fig. 6a and Supplementary Table 10), four with early–late (Fig. 6a and Supplementary Table 10) and 27 with metastasis (Fig. 6a and Supplementary Table 10). Of these associations, only three were identified in species that were also overall significantly differentially abundant (SMD *q* < 0.1) in the same contrast (Fig. 6a and Supplementary Tables 6 and 9), indicating that subspecies phylogenetic differentiation can be driven orthogonally to the enrichment or depletion of species inhabiting these ecosystems.

Specifically, after controlling for sex, age and study, we identified both *Lachnospira eligens* (formerly *Eubacterium eligens*) and *Eubacterium rectale* strain phylogenies as differential in CRC (Fig. 6b). Although *E. rectale* has previously been shown to exhibit distinct genetics by geographic origin^42^, we did control for study as proxy of geographic location, thus indicating that this species presents further genetic differentiation in CRC in addition to geography. The *Eubacterium* genus is generally considered health-associated^43^, although some publications have hypothesized a potential role for *Eubacterium* spp. in cancer^44^, and others indicated anti-proliferation activity in culture^45^. Genetic differentiation among *Eubacterium* strains may thus help to explain these disagreements. Among other clades, many strains associated with CRC were in uSGBs, including several from Ruminococcaceae (SGB15265, SGB15260, SGB4181) and one from Lachnospiraceae (SGB5089) (Fig. 6a) families, indicating that not yet fully identified species may contribute to the tumor microenvironment.

The strongest signals from this phylogenetic lineage analysis differentiated early and late CRC, as well as metastatic and nonmetastatic CRC (Fig. 6a). All species identified as having subclades associated with early or late CRC were also identified in the metastatic–nonmetastatic comparisons, likely indicating that stage IV is a driver of these differences, which can also be observed across the two highlighted species, *R. bicirculans* (Fig. 6d) and *Clostridium fessum* (Fig. 6c). In addition, *R. bicirculans* was a top hit in both models and was found through the gene carriage model to also carry genes in CRC (Anpan taxon-wise *q* < 0.1 and abs(estimate) > 1) (Fig. 6d). Of potential interest, we identified several genes involved in carbohydrate metabolism as having higher carriage by *R. bicirculans* strains in later stages of CRC (Fig. 6e), for which it is known that carbohydrate metabolism is altered, and hypothesized that *Firmicutes*-specific metabolism could promote tumor differentiation^46^.

## Discussion

Noninvasive, early CRC screening and the identification of consistent alterations in microbial components of the tumor microenvironment and bowel still have substantial room for improvement. Metagenomic profiling of the gut microbiome was shown to be highly useful for both tasks. Here, we expanded on previous metagenomic work^12–20,28^ to: (1) improve the accuracy and generalizability of metagenomic-based classifiers across populations; (2) identify additional relevant microbial biomarkers of tumor presence; (3) assess how tumor stage and other clinical variables are linked with specific microbiome configurations; and (4) investigate whether and how strain-level microbial features are linked with tumor presence and stage. By leveraging a total of 3,741 samples from 18 cohorts and applying new strain-level computational methodologies, our study has power and resolution to assess these clinically relevant outcomes.

Reproducibility of microbiome signatures in new cohorts and populations is particularly relevant for clinical screening. Based on our results, new cohorts should expect to observe an AUC of ~0.85 in CRC classification based on metagenomics, and this baseline value will further improve as more diverse and larger datasets are incorporated and as uncharacterized species are profiled by sensitive taxonomic profiling approaches^25^. We found all five SGBs assigned to the *F. nucleatum* species to be more abundant in CRC than controls, namely *F. nucleatum* subsp. *animalis*, *vincentii*, *nucleatum*, *polymorphum*, and a second SGB of *vincentii*, in decreasing order of association strength. This was in addition to other well-characterized CRC-associated microbes such as *P. micra* and *B. fragilis*. We also identified 19 additional uncharacterized SGBs with neither cultivated strains nor taxonomically defined species, highlighting a more complex CRC-associated microbial signature than previously appreciated. Our study also demonstrates that the in situ primary tumor is linked to the usual stool CRC microbiome signature, independent of the sidedness, confirming previous evidence that the primary tumor harbors usual CRC biomarkers, such as *F. nucleatum*^47^.

Investigations of the gut microbiome changes during early, late and metastatic CRC are key to better characterizing progression along the adenoma–carcinoma sequence. Although interstage microbiome shifts along with CRC progression are not as strong as those observed between CRC and controls, we found several biomarkers for late and metastatic CRC, as well as several microbial species consistently and monotonically increasing (or decreasing) from control to precursor lesion to bona fide cancer or advanced disease. In particular, late-stage CRC was found to be enriched in oral-derived species, such as *P. micra*—already involved in stimulation of tissue invasion pathways^48^—and *H. hathewayi*, which was shown to promote intestinal cell proliferation in in vitro experiments^49^. Compared with the other stages, metastatic CRC presented a higher abundance of *Methanobrevibacter smithii*, supporting previous findings that link methane producers with stage IV CRC^15^. On primary tumor location, we found that the stool samples derived from patients with CRC originating from the right-sided and transverse colon were also consistently enriched in oral species. Because several oral microorganisms form biofilms when they accumulate in the oral cavity^50^, they may show the same capacity when they grow in the gut, which is consistent with previous observations of the tumor-free mucosa in patients with right-sided CRC^22^. Coupled with the observation that left-side originating tumors are more enriched in unclassified Clostridia species, this outcome indicates the potential for small differences in gut microbes based on the location of the primary tumor, which could be related to variation in the tumor microenvironment, and carcinogenic triggers.

Prokaryotic species are remarkably genetically and functionally diverse^26^, and part of the microbiome–CRC link may be because of differences among strains or lineages in SGBs. This study performed a comprehensive strain-level analysis for CRC and found several relevant associations, including the case of otherwise-typical gut clades exhibiting differential dominant strain genetics in CRC, as well as CRC-associated species showing increased encoding of some accessory genes. Such associations were stronger than those for genes known to be directly involved in carcinogenesis (for example, *pks island* and *fragilysin*), suggesting more prevalent shifts in microbiome composition for several genes potentially relevant to adaptation to the tumor microenvironment. Dominant strain carriage was particularly associated with CRC stages, with many species (27 of the 213 tested) having significant phylogenetic associations with metastatic disease, all of which were independent of significant species-level abundance changes during CRC. Although even larger investigations are needed to assess the extent to which these strain associations are clinically relevant, they provide targeted potential mechanisms to be validated.

We found several common patterns across multiple lines of investigation. These included the role of orally derived bacteria in shaping the gut microbiome in CRC, as previously observed at lower resolution^18^. We not only strengthened the notion that the number and cumulative abundance of orally derived species are significantly higher in CRC samples than controls and adenomas, but also found that later stages of CRC were particularly enriched for oral species. Similarly, to a lesser extent, patients with right-sided CRC presented a higher number of oral-typical commensals in the gut than left-sided CRC individuals. However, many additional nonoral bacteria were also associated with CRC, including those that have been previously associated with high cardiometabolic risk^36^. Interestingly, both adenoma and later cancer stages were enriched in species linked with poor cardiometabolic health and immune-mediated diseases, possibly indicating a role for such species as proinflammatory risk factors in CRC.

Despite reported evidence that microbiome changes along CRC stages act more like a continuum than as discrete and highly differentiating configurations, we still lack better characterization of the gut microbiome significantly associated with single-stage transition and of its potential impact on the development of distant metastasis. The increased availability of stool-based metagenomic screening tests can be further exploited in new large cohorts to also improve the detection of early-phase microbiome changes occurring in this transition. Our data suggest that translation into clinical application is an option ready to be explored.

Our study has some limitations in respect to being an association-based study, thus limiting our conclusions in determining any causal relationship between microbiome configurations and tumor progression and onset, for which, however, independent evidence has been reported^10,11^.

Overall, our study reinforces the robust identification of microbiome biomarkers that can be used in stool-based screening strategies and identifies compositional and structural characteristics of the microbiome associated with disease progression to be prioritized for mechanistic studies.

## Methods

### Description of the cohorts sequenced by this study

In this study, we performed a pooled analysis expanding the set of publicly available gut microbiome sporadic CRC cohorts with six newly collected and sequenced in house cohorts (cohort 1, 2, 3, 4, 5 and 6), for a total of 1,625 new shotgun gut metagenomes. In total, 555 such new microbiome samples were collected, consistently sequenced and profiled under the ONCOBIOME Consortium, a European effort to unravel associations between intestinal microbiome alterations and different cancer types (https://www.oncobiome.eu). Samples from cohort 5 derived from a subpopulation of the NHSII^24,51^, and cohort 6 includes CRC samples and controls collected at the Umraniye Training and Research Hospital and the Department of Medical Biology, Yeditepe University (Istanbul, Turkey).

Cohort 1 includes stool samples from 163 Italian patients with stage IV CRC enrolled in a multicenter phase II clinical trial (AtezoTRIBE, collected in the University Hospital of Pisa, Italy, NCT number: NCT03721653). Cohort 2 and cohort 3 (COLOBIOME and IIGM-CZ) comprise fecal samples from the Czech Republic collected by two different research institutes (Masaryk University in collaboration with Masaryk Memorial Cancer Institute in Brno and Institute of Experimental Medicine in Prague; *n* = 204 and 124, respectively). Cohort 4 expands a previous Italian cohort at IIGM^18^ with 101 new samples. Cohort 5 includes fecal samples from 448 healthy individuals, 435 patients with adenoma and 14 patients with CRC from NHSII. Cohort 6 includes 18 patients with CRC and 39 control individuals from the Umraniye Training and Research Hospital and the Department of Medical Biology, Yeditepe University (Istanbul, Turkey). Public data considered here include four cohorts from China^16,52–54^, and eight cohorts from Austria, France, Germany, India, Italy, Japan, Spain and the United States^12–15,17–19,55^, respectively (‘Data Availability’).

In total, we considered 1,471 CRC samples, 1,191 of which have detailed information about the stage of the disease. The combined dataset comprises 94 stage 0, 253 stage I, 257 stage II, 286 stage III, 301 stage IV CRC cases (for 280 CRC samples staging was not available). In addition, 344 stool samples derived from patients with right-sided CRC, and 645 from patients with left-sided CRC. A detailed description of the cohorts included in this study is provided in the following sections and in Supplementary Table 1.

Tumor staging was defined based on the TNM and AJCC systems^56^. The TNM system measures via a three-index method the amount of growth and spreading of a tumor in a patient. In particular, it accounts for growth of the tumor to the intestinal wall or nearby organs, with no invasion of lymph nodes (T), the amount of invasion of regional lymph nodes (N) and metastases (M) in distant sites. When TNM was available, we converted it to stage, namely stage 0, stage I, stage II, stage III and stage IV^56^. We then considered stage 0–II as early-stage CRC and stage III–IV as late-stage CRC. In addition, we refer to stage IV CRC as metastatic CRC. CRC was categorized based on primary tumor location in two main classes: right-sided CRC, namely originating from the cecum, ascending colon, hepatic flexure and transverse colon; and left-sided CRC, namely originating from the splenic flexure, descending or sigmoid colon, rectosigmoid junction and rectum^57^.

#### Cohort 1 of this study: AtezoTRIBE

AtezoTRIBE (NCT03721653) is a prospective phase II clinical trial to study upfront systemic regimens in patients with unresectable stage IV CRC. Patients were not subjected to any other treatment at the time of the first stool sample collection. In total, we analyzed 56 patients presenting right-sided CRC and 91 with left-sided CRC. A further 16 samples with uncertain tumor location were considered only to study microbial trends in stages of CRC analysis. Sample collection followed the same procedure as described for cohort 3.

AtezoTRIBE is a multicenter study and the protocol was approved by the ethics committees at each participating center. The study was conducted in accordance with the Declaration of Helsinki and the International Conference on Harmonisation Guidelines for Good Clinical Practice. Participants gave written informed consent before enrollment.

#### Cohort 2 of this study: COLOBIOME

Patients were enrolled at Masaryk Memorial Cancer Institute (Brno, Czech Republic) from 2015 to 2019, as reported previously^21^. Patient inclusion criteria were: (1) scheduled for resection based on preliminary screening (such as a colonoscopy), (2) no neoadjuvant treatment, (3) no previous CRC diagnosis and (4) with confirmed stage 0–IV CRC without multiplicities (single tumor). Stool samples were collected from untreated patients before the scheduled surgery. Patients performed the collection at home, the morning of their hospitalization for the surgery, using DNA-free cotton swabs (Deltalab) and brought the samples to the hospital, where they were immediately frozen at −80 °C until further processing. In total, this cohort comprises 2 adenomas, 8 stage 0, 42 stage I, 66 stage II, 58 stage III, and 27 stage IV CRC samples. Sixty-four samples derived from individuals affected with CRC primary location in the cecum or ascending colon, 21 from individuals with CRC primary location in the transverse colon and 107 from individuals with CRC primary location in the splenic flexure descending, sigmoid, rectosigmoid or rectum. Patients provided written informed consent according to the Declaration of Helsinki.

#### Cohort 3 of this study: IIGM-CZ

Stool specimens and clinical and demographic data were collected from 124 Czech individuals recruited in two hospitals in Prague and one in Plzen, Czech Republic^58^. The individuals included in this study, like those of cohort 4, were not included in a CRC screening program, but because they were considered at risk for CRC and thus recommended to have a colonoscopy test. Based on colonoscopy results, participants were divided into: (1) 59 patients with CRC; (2) 19 patients with colorectal adenoma (13 nonadvanced and 6 advanced adenomas; no serrated lesions were collected); and (3) 38 colonoscopy-negative individuals and with 8 hyperplastic polyps^58^. All the samples from CRC cases were collected at diagnosis, before any treatment.

Naturally evacuated fecal samples were obtained from all participants previously instructed to self-collect the specimen at home. Stool samples were collected in nucleic acid collection and transport tubes with RNA stabilizing solution (Norgen Biotek) and returned to the endoscopy unit. Patients performed the collection at home before their hospitalization for the surgery and brought the samples to the hospital, where they were immediately frozen at –80 °C until DNA extraction.

#### Cohort 4 of this study: ONCOBIOME IIGM-IT

This cohort expands our previously published cohort (cohort 1 in ref. ^18^) and comprises 181 stool samples from Clinica S. Rita, Vercelli, Italy, of which 59 were from controls, 36 were from patients with adenomas and 86 were CRC samples (2 stage 0, 16 stage I, 25 stage II, 30 stage III and 5 stage IV)^58^. Among the CRC cases, 30 had tumors originating from the right colon, 6 from the transverse and 49 from the left colon or rectum. All the samples were from sporadic CRC cases, collected at diagnosis before any treatment. Samples were collected in the same way as cohort 3.

The local ethics committees of Azienda Ospedaliera SS. Antonio e Biagio e C. Arrigo of Alessandria (Italy, protocol no. Colorectal miRNA CEC2014), AOU Città della Salute e della Scienza di Torino (Italy), the Institute of Experimental Medicine of Prague (Czech Republic), Masaryk Memorial Cancer Institute (protocol no. 2018/865/MOU) and Masaryk University of Brno (Czech Republic, protocol no. EKV2019-044) approved the study (cohorts 2, 3 and 4). All patients gave written informed consent following the Declaration of Helsinki before participating in the study.

#### Cohort 5 of this study: NHSII

NHSII is a cross-sectional, prospective study of CRC-related gut microbial composition. The study protocol was approved by the institutional review boards of the Brigham and Women’s Hospital and Harvard T.H. Chan School of Public Health, and those of participating registries as required. Participants provided written informed consent before study enrollment and stool collection. Specifically, this study recruited a subpopulation of NHSII^24,51^, a long-running prospective cohort from the United States. All participants contributed a stool sample, with the research aiming to investigate the role of the gut microbiome specifically in participants with recent adenomas. The adenoma (*n* = 435) and CRC cases (*n* = 14) in the study were one-to-one matched with healthy control samples (*n* = 448) based on age at stool collection, ethnicity, month of collection, state of residence and total number of, reason for and date of recent endoscopy. For a subset of cases (*n* = 39) the matching criteria for ethnicity (expanded definition of Caucasian) and age at collection were relaxed. Adenomas were further defined as high or low risk based on the location, number and histology of the cells. The CRC samples ranged from all stages and were limited as only a limited number of participants in NHSII have been diagnosed with CRC and recently contributed a stool sample. Previously stored, Genotek’s OMNIgene fixed, and −80 °C frozen stool samples were processed and sequenced for shotgun metagenomics at Diversigen.

#### Cohort 6 of this study: Turkish CRC cohort

Patients with CRC were recruited at the Umraniye Training and Research Hospital while healthy volunteers contributing to science used as controls were recruited at the Department of Medical Biology, Yeditepe University (both in Istanbul, Turkey). Naturally evacuated fecal samples were obtained from subjects previously instructed to self-collect the specimen at home. For patients with CRC, collection was performed before surgical resection. Samples from participants who had used antibiotics within 1 month before the sample collection were excluded. Samples were collected in nucleic acid collection and transport tubes with RNA stabilizing solution (Norgen Biotek). Stool aliquots (200 μl) were stored at −80 °C until RNA extraction.

The study was approved by the ethics committee of the Umraniye Training and Research Hospital, Istanbul Turkey (ref no. 351, 19/11/2020).

### ONCOBIOME sample sequencing and preprocessing

DNA was extracted from stool samples with the DNeasy PowerSoil Pro Kit (Qiagen), and sequencing libraries were prepared using the Illumina DNA Prep, (M) Tagmentation kit (Illumina), following the manufacturer’s guidelines. The library pool was subjected to a cleaning step with 0.7× Agencourt AMPure XP beads. Samples were sequenced on a NovaSeq 6000 S4 flow cell (Illumina) at the University of Trento sequencing facility. Sequenced metagenomes were preprocessed using the pipeline available at https://github.com/SegataLab/preprocessing for: (1) removal of low-quality reads (quality <20), too short fragments (length <75 bp), and reads with two or more ambiguous nucleotides; (2) host contaminant DNA removal using Bowtie 2 (ref. ^59^) (--sensitive-local) for the phiX174 Illumina spike-in and human-associated reads (hg19); and (3) creation of paired forward and reverse and unpaired reads output files. Once preprocessed, ONCOBIOME samples presented an average of 37 million reads.

### Cohort 6 sequencing and preprocessing

Stool DNA extraction and library preparation followed the procedure described for the ONCOBIOME cohorts. A final clean-up of the library pool was performed with 0.6× AMPure XP beads (Beckman-Coulter), and then resuspended with one-third of the initial pool volume. Sequencing was performed with a NovaSeq 6000 at the IIGM sequencing facility. Cohort 6 was preprocessed with the same pipeline used for the ONCOBIOME studies.

### NHSII sample sequencing and preprocessing

For DNA extraction and sequencing, samples were sent to Diversigen and all steps were completed according to their standardized DEEPSEQ protocol. Briefly, samples were extracted with the PowerSoil Pro (Qiagen) kit using the automated high-throughput method on the QiaCube HT (Qiagen). This used Powerbead Pro Plates (Qiagen) with 0.5 and 0.1 mm ceramic beads, but otherwise followed the manufacturer’s protocol. DNA amount and quality were assessed with a Quant-iT PicoGreen dsDNA Assay (Invitrogen) post extraction. Libraries were prepared with a modified protocol from the Nextera Library Prep kit (Illumina) and sequenced on an Illumina NovaSeq using paired-end 2 × 150 reads (Illumina). Sequenced samples were then filtered for host contamination via the KeandData pipeline (https://github.com/biobakery/kneaddata). In particular, this pipeline consists of three main steps: a first trimming of poor-quality reads with trimmomatic^60^, specifically we applied a sliding window trim removing reads after four subsequent bases had a Phred score of 20 or less, and then reads with fewer than 60 base pairs were removed. Next, we filtered repetitive reads with the tandem repeats finder^61^, and removed adapters with trimmomatic. Finally, host and common sequencing components decontamination was completed with Bowtie 2 against PhiX and the human genome (hg37).

### Taxonomic, functional and strain-level profiling

We applied MetaPhlAn 4 (v.4.0.0, database vJan21, with the ‘--statq 0.1’)^25^ and HUMAnN 3.6 (ref. ^28^) profiling tools to produce microbial taxonomic and functional profiles, respectively. In addition, StrainPhlAn 4 (v.4.0.3)^25^ was run to generate dominant single nucleotide variant profiles for any species that passed the filtering steps in StrainPhlAn (213; species are filtered for sufficient markers and samples to run the tool). Newly sequenced samples and public data considered in this study were profiled consistently.

### Public CRC gut microbiome studies

We considered metagenomic samples from 11 public CRC–control studies, 8 of which had already been included in previous meta-analyses^18,19^. Metadata for these cohorts were available in the curatedMetagenomicData^27^ package and the metagenomes were available in the Sequence Read Archive (SRA) or the European Nucleotide Archive (ENA) with the following accession codes: PRJEB7774 for Feng, Q. (2015)^12^; PRJNA531273, PRJNA397112 for Gupta, A. (2019)^13^; metagenomic data for Obón-Santacana, M. (2022)^55^ was requested from the authors of the study; PRJNA447983 for Thomas, A.M. (2019)^18^; PRJEB12449 for Vogtmann, E. (2016)^14^; PRJEB27928 for Wirbel, J. (2019)^19^; DRA006684 and DRA008156 for Yachida, S. (2019)^15^; PRJEB10878 for Yu, J. (2017)^16^; and PRJEB6070 for Zeller, G. (2014)^17^. Metagenomic samples for three additional public studies (Liu, N.N. (2022)^54^, Yang, J. (2020)^52^ and Yang, Y. (2021)^53^) were available in the European Nucleotide Archive (ENA) (accession numbers: PRJNA731589, PRJNA429097 and PRJNA763023, respectively).

#### Feng, Q. (2015)

This cohort^12^ comprises 154 Austrian individuals (61 controls, 47 adenomas and 46 CRC). Staging was available for 45 CRC (7 stage 0, 17 stage I, 9 stage II, 11 stage III and 1 stage IV), with 8 right-sided CRC and 38 left-sided CRC. Patients did not receive antibiotics in the 3 months before collection of the stool sample.

#### Gupta, A. (2019)

This cohort^13^ includes 60 stool samples from the same number of individuals from India (equally distributed between Bhopal and Kerala) divided into 30 controls and 30 CRC cases. All the fecal samples in this cohort were collected from people who were not subject to antibiotics close to the sampling date and had not been diagnosed with other diseases.

#### Liu, N.N. (2022)

This cohort^54^ comprises 164 stool samples from an equal number of individuals from China (85 controls and 79 CRC cases). The cohort derives from the ‘Chinese cohort in Shanghai (CHN_SH)’, whose patients were sampled after CRC diagnosis and before any treatment. All the cases include exclusively sporadic CRC. Control individuals were recruited in the Taizhou Imaging Study. Age, sex, body mass index (BMI) and case or control condition were retrieved from the original publication and the corresponding ENA portal project (PRJNA731589).

#### Obón-Santacana, M. (2022)

This cohort^55^ includes a subset of the participants in the COLSCREEN study. In total, 156 participants were selected (51 controls, 54 high-risk lesions and 51 CRC) and stool samples collected. Participants were asked to collect the stool sample 1 week before colonoscopy preparation and participants who reported use of antibiotics or probiotics within 1 month before sample collection were excluded.

#### Thomas, A.M. (2019)

This is ‘cohort 2’ of the study^18^ and comprises 60 stool samples, collected from the same number of individuals recruited at the European Oncology Institute in Milan, Italy. In particular, the cohort consisted of 28 controls and 32 CRC cases for which no staging or primary tumor location information was available. No subjects reported antibiotic use in the 6 months before the sampling. For CRC cases, sampling was performed before surgery or any cancer treatment.

#### Vogtmann, E. (2016)

In total, 110 stool samples were collected from an equal number of US individuals divided into 58 controls and 52 CRC cases (12 stage II, 21 stage III and 18 stage IV; 15 right-sided CRC, 32 left-sided CRC). CRC samples were collected before surgery or any other cancer treatment^14^.

#### Wirbel, J. (2019)

This cohort^19^ comprises 125 stool samples, collected from the same number of German individuals. The cohort included 65 controls and 60 CRC cases (3 stage 0, 15 stage I, 20 stage II, 10 stage III and 12 stage IV; 15 right-sided CRC and 42 left-sided CRC). CRC samples were recruited in the ColoCare study and fecal samples were collected after colonoscopy. Control samples were recruited in the PRÄVENT study.

#### Yang, J. (2020)

This cohort^52^ includes 193 stool samples, collected from the same number of individuals and included 95 controls and 98 CRC cases from China (23 stage I, 36 stage II, 31 stage III and 8 stage IV). Stool samples were excluded if individuals used antibiotics, were subjected to radiotherapy or corticosteroids in the month before the sampling. Age, sex, case or control, TNM, stage and primary location were obtained from the original publication and the corresponding ENA portal project (PRJNA429097).

#### Yang, Y. (2021)

This cohort^53^ comprises 200 stool samples from the same number of individuals from the Fudan cohort (China) and includes 100 controls and 100 CRC cases. Only samples from individuals who did not use antibiotics or probiotics for 1 month before recruitment were included in the study. CRC stool samples were collected before colonoscopy or other cancer therapies and surgery. Only sporadic CRC cases were included, with no history of inflammation-associated CRC, intestinal bowel syndrome or other cancers. Disease categories (CRC or control) were retrieved from the original publication; raw metagenomes were obtained from the ENA portal accession number PRJNA763023.

#### Yachida, S. (2019)

This study^15^ comprises 616 stool samples from the same number of Japanese individuals subjected to colonoscopy. The cohort included 291 controls, 67 adenomas, and 258 CRC cases (73 Stage 0, 75 Stage I, 36 Stage II, 52 Stage III, and 22 Stage IV; 83 right-sided CRC, and 167 left-sided). Only sporadic CRC cases were considered, with no inflammatory bowel disease (IBD) or abdominal surgical history.

#### Yu, J. (2017)

This cohort^16^ comprises 128 samples from the same number of individuals, collected in Hong Kong, China, and included 53 controls and 75 CRC cases (12 Stage I, 24 Stage II, 24 Stage III, and 8 Stage IV; 11 right-sided and 54 left-sided CRC).

#### Zeller, G. (2014)

This cohort^17^ includes stool samples from 156 individuals from France, with 61 controls, 42 adenomas, 53 CRC cases (15 Stage I, 7 Stage II, 10 Stage III, and 21 Stage IV; 17 were right-sided CRC and 36 were left-sided CRC). Samples were collected before colonoscopy.

### Definition of oral-typical SGBs

For the definition of the oral signature, we collected the data available from 5 datasets for a total of 495 healthy individuals for whom both stool and oral (either from saliva or tongue dorsum) samples were available for the same subject and time point. The identified datasets are: BritoIL_2016 (116 participants, stool and saliva)^62^, FerrettiP_2018 (20 participants, stool and tongue dorsum)^63^, HMP_2012 (85 participants with stool and tongue dorsum samples)^31^, KartalE_2022 (39 participants, stool and saliva)^64^ and NagataN_2022 (235 participants, stool and saliva)^65^. The oral signature was defined based on the distribution of specific microbial species that met the following criteria: (1) present exclusively in the oral cavity of at least 20% of participants; (2) found in both the oral cavity and stool of fewer participants than those that were exclusively oral; and (3) present exclusively in stool in fewer than 5% of participants. These constraints resulted in a signature of 235 oral-typical species (Supplementary Table 3).

### Alpha*-* and beta-diversity

To assess alpha-diversity we used the Shannon index (vegan R package) and the richness computed as the number of species present in a sample. Dimensionality reduction was been performed using the Rtsne function from the Rtsne R package, providing the Bray–Curtis dissimilarity matrix as input. PERMANOVA was performed using the adonis2 function from the vegan R package with 999 permutations and blocked for study of origin by the setBlocks function, with and without including age, sex and BMI in the model. The distance matrix used both for the multidimensional scaling and the PERMANOVA is based on the Bray–Curtis dissimilarity matrix estimated with the vegdist function from the vegan R package. We reported *R*^2^ for each test. Comparisons with *P* ≤ 0.01 were considered significant.

### Oral-to-gut SGBs quantification

To quantify gut colonization by typically oral commensal species (defined in the previous section), we developed two quantitative scores. The ‘oral-to-gut score’ for each stool sample sums the relative abundance of the oral SGBs present. The ‘oral-to-gut richness,’ in contrast, counts the number of distinct oral SGBs present in each stool sample.

### Meta-analysis

Because this work is a multicohort study and a batch effect exists in data from different origins, we used the meta-analysis of SMDs computed in each dataset instead of effect sizes computed from batch-effect corrected data as the primary approach for biomarker discovery. Our choice is motivated by the fact that correcting for batch effect is a difficult task, because of both incomplete information on batch effects (not only between cohorts, but also within cohorts), and the lack of a consensus approach for batch correction in microbiome studies. In particular, SMDs were computed with Hedges’ method^66^ which adds a correction for low sample bias to Cohen’s *d* estimator. Meta-analysis was performed using the metacont function from the meta R package. Between-study variance (𝜏^2^) was estimated via restricted maximum likelihood and CIs of the summary effect were adjusted with the Hartung and Knapp method^67^. This procedure was applied to all microbiome features with at least 10% prevalence and present in at least five samples in one of the testing sets when at least three studies presented a minimum of ten samples for each class. Adjusted *P* values (*q* values, in the text) were computed via the Benjamini–Hochberg procedure. Significance was determined as Benjamini–Hochberg *q* < 0.1 or *P* < 0.01. Meta-analysis of standardized linear model estimates was applied to determine the effect sizes corrected for age, sex and BMI. Per-cohort linear models were fitted for each feature relative abundance (arcsine square root transformed) with the additive effect of age, sex and BMI. Once standardized by the standard error, we performed standard meta-analysis as described above.

### Machine learning approaches

For the ML analysis, we used the random forest classifier as implemented in the metaml tool (https://github.com/SegataLab/metaml)^29^. An ensemble of 1,000 trees with a minimum of 5 samples per leaf (grid-search optimal max features per split to consider in CV, and no other normalization performed) was trained and tested in the following settings on the data: (1) per-dataset CV (10-fold CV repeated 20 times); (2) across-study prediction (for each pair of studies the classifier is trained on one and tested on the other); and (3) LODO approach (each cohort becomes the testing set while all the others are used for training). CV comparisons were considered when presenting at least 15 samples for each class in one cohort, whereas between-dataset CV and LODO comparisons were considered when 15 samples were available in each class both in the training and validation sets. NHSII was included in the training set for comparison of controls versus CRC in LODO, but was not considered a validation cohort for the unbalanced sample sizes between the two classes. This setup was extensively applied in previous works^18,28^, allowing for robust comparison of our results with those in the literature. Relative abundance profiles (values in the [0, 1] range) were previously arcsine square root transformed. When testing for oral-typical or non-oral species, after selection, we rescaled the relative abundance to [0,1], and then transformed via arcsine square root. No other feature selection was performed otherwise.

For the reasons mentioned above, we decided not to integrate the studies in a single large cohort and perform batch-effect correction, previous to ML. Our approach treats each study independently and tests the strength of the trained model in the same study (per-dataset CV), in a different study (across-studies prediction) or in the left-out study when validated in LODO. This ensures that batch-effect correction does not introduce favorable bias in the classification tasks.

### Linear mixed model (MaAsLin 2)

Linear mixed models, via the MaAsLin 2 R package^68^, were iteratively applied fitting each microbial abundance profile (after arcsine square root transformation) with sample condition (control, adenoma, CRC stage 0, I, II, III, IV) as the fixed effect, and originating cohort as the random effect. The MaAsLin 2 ‘LM’ model uses the lmer function from the lmerTest R package. Correction of the *P* values of the coefficients from the models was performed using the Benjamini–Hochberg procedure, as obtained from MaAsLin 2. For the fitting of each model, species were considered if they were at least 10% prevalent across all controls, adenoma and stages 0–IV. Concordant signature between stages was computed by applying the Jaccard similarity, which consists of the number of elements in the intersection between the signatures deriving from each stage, divided by the number of elements in the union between them. We applied MaAsLin 2 with the same setting to test SGB differential abundant between primary tumor locations in stage IV CRC. In both analyses, only associations with *q* < 0.1 were considered significant.

### Strain-level analysis with Anpan

To complete subspecies clade-level association analysis with CRC, we used Anpan (v.0.3.0, https://huttenhower.sph.harvard.edu/anpan)^69^, an R package that quantifies the associations between differential gene carriage, subspecies phylogenetic structure and host phenotypic outcomes. The gene model in Anpan addresses two key issues: robust and accurate detection of species whose genes are well-covered in shotgun metagenomes; and sensitive detection of consistently associated genes with phenotypic outcomes. Anpan first filters samples to remove any without enough species-specific gene coverage to accurately assess gene-level effects. A GLM was then used to model each gene’s association with the outcome (accounting for metadata covariates), followed by FDR correction. Here, our outcomes of interest were CRC or control, early- or late-stage CRC and primary tumor location (right-sided versus left-sided), adjusted for age, sex and study (which accounts for the geographic location of collection).

From the predicted genes with Anpan, we first quantified the number of significant hits per species, with a significance threshold of an absolute coefficient of 2 and *q* < 0.05. Next, we regrouped the UniRef90 genes to GO terms by direct matching. We also used annotations from FUGAsseM to add predicted GO term annotations based on metagenomic and metatranscriptomic covariation patterns. Specifically, FUGAsseM predicts the functions of uncharacterized gene products in the context of microbial communities by integrating multiple types of community-wide evidence. It extends ‘guilt by association’ approaches by building an individual random forest classifier predicting gene function based on each data type, followed by an ensemble tier that builds an integrated classifier combining the learning results from the first tier. As a result, putative functional annotations are assigned to uncharacterized proteins that achieve high prediction probability.

Next, we assessed the phylogenetic associations with our outcomes of interest. StrainPhlAn trees (http://segatalab.cibio.unitn.it/tools/strainphlan/) were used as input to phylogenetic generalized linear mixed models to assess phylogenetic associations with the outcomes. Phylogenetic generalized linear mixed models are probabilistic models that account for phylogenetic structure by encoding the tree structure as a correlation matrix. For these models, we also used age and sex as covariates and study as an offset variable. We referred to associations as hits if the phylogenetic model improved the ELPD over a base GLM (the same model except without phylogenetic information) by more than 2. ELPD is a model comparison metric akin to the Akaike information criterion.

### Strain-level feature identification

We tested whether the inclusion of strain-related microbial features in a PERMANOVA leads to improvement in the model association or prediction. Because phylogenetic information was already tested via anpan, we developed a complementary approach for defining strain-level features based on strain preferences for a given nucleotide in marker genes. In particular, starting from StrainPhlAn 4 reconstructed multiple sequence alignments of the marker genes for the 213 SGBs, we selected genetic positions in marker genes with binary entropy at least 0.5 (to remove positions with little strain-level variability), by selecting those that presented the minimum number of gaps in clusters of 1-ANI (average nucleotide identity) ≤0.05 (to remove features very correlated to each other). We then expanded each position into five features using one-hot encoding, one for each nucleotide and one representing a gap. The value in each of these features can be either 1 when the corresponding nucleotide or gap is present in that position, or 0 otherwise. In this way, we obtained a total of 1,382,825 features across all the SGBs. Given the large number of features produced, we then applied an additional set of thresholds based on prevalence and removing collinear ones. In particular, features with <20% prevalence or prevalence >80% in the controls and CRC samples were removed, ensuring that very rare or too common base preferences were not considered, thus obtaining 42,094 features. We then removed features highly correlated (Pearson correlation >0.5) with any other feature, selecting the first occurrence as the representative and discarding all other features that correlated with it with a higher absolute Pearson coefficient than the threshold selected (0.5). This step produced a set of 2,722 features for the 0.5 Pearson correlation threshold. PERMANOVA tests with this feature set were performed as described earlier in Methods, specifying Jaccard as the distance measure.

### Estimation of cardiometabolic microbial signature from the PREDICT 1 study

The cardiometabolic microbial signature was estimated, as reported in our previous work^25,36^, as the species most associated with the set of cardiometabolic indices defined in ref. ^36^. In brief, partial Spearman correlations were computed between each SGB and the set of indices associated with cardiometabolic risk, correcting for sex, age and BMI. Partial correlations were ranked and averaged first in each category and then across categories to derive a global rank. Ranks ranged between 0 and 1 for the most favorable and unfavorable species, respectively, and we considered those SGBs with a rank above the third quartile of the distribution. We identified 115 SGBs representing a higher cardiometabolic risk and that account for 2.97% of the detected SGBs across all analyzed cohorts.

### Definition of the signature for cardiovascular disease, T2D, IBD and inflammatory diseases

We compared the signatures found for CRC in meta-analysis with signatures for other disease types or groups or diseases. Specifically, we searched in the curatedMetagenomicData 3 repository for case-control studies for T2D (control *n* = 882, cases *n* = 750, four cohorts)^70–73^, ulcerative colitis (*n* = 247 and 84), CD (*n* = 291 and 83, three cohorts in total)^38,74,75^, inflammatory diseases (including asthma, Behcet syndrome, multiple sclerosis, rheumatoid arthritis and myalgic encephalomyelitis, *n* = 918 and 827, five cohorts)^76–80^. IBD was obtained with a quarry of ulcerative colitis and CD. We profiled their reads with MetaPhlAn 4. Then, to compare the microbial signature associated with CRC, we arcsine square root transformed all the MetaPhlAn 4 SGB-level relative abundances, we performed a meta-analysis of SMDs computed starting from a linear regression linking the disease state to the SGB transformed abundance and adjusted by country to take into account potential population effect. SMDs and uncertainty estimations were meta-analyzed via inverse variance weighting using Paule–Mandel heterogeneity. From the resulting tables, signatures for the six disease types were retrieved by selecting those SGBs having an FDR for the meta-analysis *P* value <0.1 and being found in a minimum of three datasets.

### Reporting summary

Further information on research design is available in the Nature Portfolio Reporting Summary linked to this article.

## Online content

Any methods, additional references, Nature Portfolio reporting summaries, source data, extended data, supplementary information, acknowledgements, peer review information; details of author contributions and competing interests; and statements of data and code availability are available at 10.1038/s41591-025-03693-9.

## Supplementary information

Reporting Summary

Supplementary Tables Supplementary Tables 1–10.

## Data Availability

Stool metagenomes’ sequences for the four new ONCOBIOME cohorts are available in the European Nucleotide Archive (ENA) with the project numbers PRJEB72524, PRJEB72525, PRJEB72526 and PRJEB72523. The NHSII cohort is available in NCBI Sequence Read Archive (SRA) with the project id PRJNA1237248. Metagenomic sequences for Cohort 6 are available in NCBI via the project number PRJNA1167935. MetaPhlAn 4 and HUMAnN 3.6 profiles and metadata for the cohorts included in this study are available via *Z*enodo at 10.5281/zenodo.15069069 (ref. ^81^).

## Code availability

No original tool was developed for this manuscript. All the applied approaches have been previously published or are publicly available and referenced in the manuscript. All the code used for the analysis is available in this GitHub folder: https://github.com/SegataLab/CRC_staging_analysis.
