# Supplementary material for: Pooled analysis of 3,741 stool metagenomes from 18 cohorts for cross-stage and strain-level reproducible microbial biomarkers of colorectal cancer
Source: Nat Med. 2025 Jun 3;31(7):2416–29. doi: 10.1038/s41591-025-03693-9 (PMC12283368; doi:10.1038/s41591-025-03693-9)
Supplement: Supplementary file 1 — Reporting Summary [file 41591_2025_3693_MOESM1_ESM.pdf]

nature portfolio

Corresponding author(s): Nicola Segata

Last updated by author(s): Mar 24, 2025

Reporting Summary

Nature Portfolio wishes to improve the reproducibility of the work that we publish. This form provides structure for consistency and transparency in reporting. For further information on Nature Portfolio policies, see our Editorial Policies and the Editorial Policy Checklist.

Statistics

For all statistical analyses, confirm that the following items are present in the figure legend, table legend, main text, or Methods section.

n/a	Confirmed	
☐	☒	The exact sample size (n) for each experimental group/condition, given as a discrete number and unit of measurement
☐	☒	A statement on whether measurements were taken from distinct samples or whether the same sample was measured repeatedly
☐	☒	The statistical test(s) used AND whether they are one- or two-sided<br>*Only common tests should be described solely by name; describe more complex techniques in the Methods section.*
☐	☒	A description of all covariates tested
☐	☒	A description of any assumptions or corrections, such as tests of normality and adjustment for multiple comparisons
☐	☒	A full description of the statistical parameters including central tendency (e.g. means) or other basic estimates (e.g. regression coefficient) AND variation (e.g. standard deviation) or associated estimates of uncertainty (e.g. confidence intervals)
☐	☒	For null hypothesis testing, the test statistic (e.g. F, t, r) with confidence intervals, effect sizes, degrees of freedom and P value noted<br>*Give P values as exact values whenever suitable.*
☒	☐	For Bayesian analysis, information on the choice of priors and Markov chain Monte Carlo settings
☒	☐	For hierarchical and complex designs, identification of the appropriate level for tests and full reporting of outcomes
☐	☒	Estimates of effect sizes (e.g. Cohen's d, Pearson's r), indicating how they were calculated

Our web collection on statistics for biologists contains articles on many of the points above.

Software and code

Policy information about availability of computer code

Data collection	No commercial code has been used for data collection.
Data analysis	All the analysis in the paper has been performed with open source software, as extensively described in the Methods section of the manuscript. The code used is deposited on GitHub (https://github.com/SegataLab/preprocessing for the preprocessing pipeline, https://github.com/SegataLab/metaml for metaml, and https://github.com/SegataLab/CRC_staging_analysis for the custom scripts used in the manuscript). These are versions of the tools and packages used in the paper: R version 4.2.2, vegan 2.6-4, meta 7.0.0, MaAsLin 2. Microbial profiling was performed with MetaPhlAn 4 (version 4.0.0, database vJan21, , with the "--statq 0.1") and HUMAnN 3.6. Strain-level profiling was performed via StrainPhlAn 4 (version 4.0.3) and the analysis with Anpan (v0.3.0).

For manuscripts utilizing custom algorithms or software that are central to the research but not yet described in published literature, software must be made available to editors and reviewers. We strongly encourage code deposition in a community repository (e.g. GitHub). See the Nature Portfolio guidelines for submitting code & software for further information.

Data

Policy information about availability of data

All manuscripts must include a data availability statement. This statement should provide the following information, where applicable:

- Accession codes, unique identifiers, or web links for publicly available datasets
- A description of any restrictions on data availability
- For clinical datasets or third party data, please ensure that the statement adheres to our policy

Stool metagenomes' sequences for the four new ONCOBIOME cohorts are available in the European Nucleotide Archive (ENA) with the project numbers PRJEB72524, PRJEB72525, PRJEB72526, and PRJEB72523. The NHSII cohort is available in NCBI Sequence Read Archive (SRA) with the project id PRJNA1237248. Metagenomic sequences for Cohort 6 are available in NCBI via the project number PRJNA1167935. MetaPhlAn 4 and HUMAnN 3.6 profiles and metadata for the cohorts included in this study are available on Zenodo (https://doi.org/10.5281/zenodo.15069069).
We considered in this work metagenomic samples from 11 public CRC/control studies, 8 of which already included in previous meta-analyses. Metadata for these cohorts was available in the curatedMetagenomicData package and the metagenomes were available either in the Sequence Read Archive (SRA) or the European Nucleotide Archive (ENA) with the following accession codes: PRJEB7774 for FengQ_2015, PRJNA531273, PRJNA397112 for GuptaA_2019, metagenomic data for ObónSantacanaM_2022 was requested to the authors of the study, PRJNA447983 for ThomasAM_2018, PRJEB12449 for VogtmannE_2016, PRJEB27928 for WirbelJ_2018, DRA006684 and DRA008156 for YachidaS_2019, PRJEB10878 for YuJ_2015, PRJEB6070 for ZellerG_2014. Metagenomic samples for three additional public studies (LiuNN_2022, YangJ_2020 and YangY_2021) were available in the European Nucleotide Archive (ENA) (accession numbers: PRJNA731589, PRJNA429097, and PRJNA763023, respectively).

Research involving human participants, their data, or biological material

Policy information about studies with human participants or human data. See also policy information about sex, gender (identity/presentation), and sexual orientation and race, ethnicity and racism.

Reporting on sex and gender	Personal information was collected at the time of sample collection for the novel cohorts presented in this study. Our results are obtained considering samples from male and female individuals together. Sex information is reported for each sample in the Supplementary Tables.<br>Targeted analysis on sex has not been performed, since these associations are not the focus of the manuscript. Sex was included as covariate in the analysis.
Reporting on race, ethnicity, or other socially relevant groupings	No socially constructed or socially relevant categorization variables are used in the manuscript.
Population characteristics	The analysis performed in this study has been performed accounting for possible confounders, such as age, sex and BMI, and stratified by study. Age in our study ranged from 20 to 90 with a median value of 64. Our study included more 1,976 female and 1,558 male individuals. BMI ranged from 11.46 to 52.25 with an average value of 25.48 (median 24.64).
Recruitment	Recruitment of participants in this study is described in details in the Methods section of the manuscript. In particular, individuals in Cohort 1 (AtezoTRIBE) were enrolled for the clinical trial, and they were not subjected to other treatment before sample collection; individuals in Cohort 2 (COLOBIOME) were enrolled in Masaryk Memorial Cancer Institute (Brno, Czech Republic) and adhered to the following inclusion criteria: were (i) scheduled for resection based on preliminary screening (such as a colonoscopy), (ii) no neoadjuvant treatment, (iii) no previous CRC diagnosis (iv) with confirmed stage 0–IV CRC without multiplicities (single tumor), and patients were not treated or subjected to surgery before the collection. Individuals from Cohort 3 (IIGM-CZ) were recruited in two hospitals in Prague and one in Plzen, Czech Republic. These individuals were not included in a CRC screening program, but because they were considered at risk for CRC and thus recommended to have a colonoscopy test. Samples from CRC cases were collected at diagnosis, before any treatment. Individuals from Cohort 4 were recruited from Clinica S. Rita in Vercelli, Italy. All the samples were from sporadic CRC cases, collected at diagnosis before any treatment, and expand our previously published cohort in Thomas et al. 2019. Individuals from Cohort 5 (NHS II) were recruited in a cross-sectional, prospective study (the NHSII) of CRC-related gut microbial composition. Individuals from Cohort 6 were recruited at the Umraniye Training and Research Hospital while healthy volunteers contributing to science used as controls were recruited at the Department of Medical Biology, Yeditepe University (both Institutes in Istanbul, Turkey). For CRC patients collection was performed before surgical resection. Samples from participants having used antibiotics within one month before the sample collection were excluded.<br><br>Samples included in the study from the novel 6 cohorts presented were selected using consistent exclusion criteria, also common to the publicly available cohorts, thus not introducing any specific bias in the analysis related to possible previous treatments or colonoscopy. Recruitment for publicly available cohorts is described in the original publications
Ethics oversight	Cohort 1: AtezoTRIBE is a multi-centre study and the protocol was approved by the Ethics Committees at each participating center. The study was conducted in accordance with the Declaration of Helsinki and the International Conference on Harmonisation Guidelines for Good Clinical Practice. Participants gave written informed consent before enrolment.<br>Cohort 2: Patients were enrolled at Masaryk Memorial Cancer Institute (Brno, Czech Republic) from 2015 to 2019, as reported previously (https://doi.org/10.3390/cancers13194799).<br>Cohort 3,4: The local ethics committees of Azienda Ospedaliera SS. Antonio e Biagio e C. Arrigo of Alessandria (Italy, protocol no. Colorectal miRNA CEC2014), AOU Città della Salute e della Scienza di Torino (Italy), the Institute of Experimental Medicine of Prague (Czech Republic), Masaryk Memorial Cancer Institute (protocol no. 2018/865/MOU), and Masaryk University of Brno (Czech Republic, protocol no. EKV2019-044) approved the study (Cohort 2, 3 and Cohort 4). All patients gave written informed consent following the Declaration of Helsinki before participating in the study.

Cohort 5: The study protocol was approved by the institutional review boards (IRBs) of the Brigham and Women's Hospital and Harvard T.H. Chan School of Public Health, and those of participating registries as required. Participants provided written informed consent before study enrollment and stool collection.
Cohort 6: The study was approved by the Ethics committee of the Umraniye Training and Research Hospital, Istanbul Turkey (Ref n. 351, 19/11/2020).

Note that full information on the approval of the study protocol must also be provided in the manuscript.

Field-specific reporting

Please select the one below that is the best fit for your research. If you are not sure, read the appropriate sections before making your selection.

☒ Life sciences ☐ Behavioural & social sciences ☐ Ecological, evolutionary & environmental sciences

For a reference copy of the document with all sections, see nature.com/documents/nr-reporting-summary-flat.pdf

Life sciences study design

All studies must disclose on these points even when the disclosure is negative.

Sample size	This study includes 3,741 stool shotgun metagenomic samples sequenced from the same number of individuals from 18 cohorts. Details about studies included and sample sizes are reported in the manuscript. Cohorts included in this study present at least 10 samples in each class of at least one of the tested comparisons (cases vs controls, right- vs left-sided tumor, early- vs late-stages CRC). This value is sufficient for defining a meaning effect size that can be evaluated in our meta-analytic approach, as well as for the machine learning evaluation.
Data exclusions	Samples from the public cohorts have been employed in the analyses without additional filtering. The samples newly sequenced for the studies have been quality checked as described in the manuscript. In particular, samples with less than 2M reads after preprocessing (removal of low quality reads and host contamination) were excluded.
Replication	For replication of the study, we are providing both the metagenomes in publicly available databases, as well as the profiles and metadata in Zenodo (https://doi.org/10.5281/zenodo.15069069) and Supplementary Tables in the manuscript.
Randomization	Covariates, such as sex, age and BMI were included in the models (e.g. random effect models) in our study. We thus presented these results alongside crude models. We demonstrate in the study that the presence of the primary tumor in the intestine is the major determinant of the CRC microbial signature, more than these other covariates.
Blinding	Information about individuals conditions was already available during the study. We overcome this limitation applying cross-study validation for the machine learning and meta-analysis and pooled analysis for determining microbial biomarkers for CRC.

Reporting for specific materials, systems and methods

We require information from authors about some types of materials, experimental systems and methods used in many studies. Here, indicate whether each material, system or method listed is relevant to your study. If you are not sure if a list item applies to your research, read the appropriate section before selecting a response.

Materials & experimental systems

n/a	Involved in the study	
☒	☐	Antibodies
☒	☐	Eukaryotic cell lines
☒	☐	Palaeontology and archaeology
☒	☐	Animals and other organisms
☒	☐	Clinical data
☒	☐	Dual use research of concern
☒	☐	Plants

Methods

n/a	Involved in the study	
☒	☐	ChIP-seq
☒	☐	Flow cytometry
☒	☐	MRI-based neuroimaging

Plants

Seed stocks	*Report on the source of all seed stocks or other plant material used. If applicable, state the seed stock centre and catalogue number. If plant specimens were collected from the field, describe the collection location, date and sampling procedures.*
Novel plant genotypes	*Describe the methods by which all novel plant genotypes were produced. This includes those generated by transgenic approaches, gene editing, chemical/radiation-based mutagenesis and hybridization. For transgenic lines, describe the transformation method, the number of independent lines analyzed and the generation upon which experiments were performed. For gene-edited lines, describe the editor used, the endogenous sequence targeted for editing, the targeting guide RNA sequence (if applicable) and how the editor was applied.*
Authentication	*Describe any authentication procedures for each seed stock used or novel genotype generated. Describe any experiments used to assess the effect of a mutation and, where applicable, how potential secondary effects (e.g. second site T-DNA insertions, mosiacism, off-target gene editing) were examined.*
